# A Systematic Review of the Effect of Dietary Supplements on Cognitive Performance in Healthy Young Adults and Military Personnel

**DOI:** 10.3390/nu12020545

**Published:** 2020-02-20

**Authors:** Diane E. Pomeroy, Katie L. Tooley, Bianka Probert, Alexandra Wilson, Eva Kemps

**Affiliations:** 1 Cognition and Behaviour, Land Division (Edinburgh), Defence Science & Technology, Department of Defence, Edinburgh, South Australia 5111, Australia; Katie.Tooley@dst.defence.gov.au; 2 Food and Nutrition, Land Division (Scottsdale), Defence Science & Technology, Department of Defence, Scottsdale, Tasmania 7260, Australia; Bianka.Probert@dst.defence.gov.au; 3 School of Psychology, Flinders University, Bedford Park, South Australia 5042, Australia; wils0602@flinders.edu.au (A.W.); Eva.Kemps@flinders.edu.au (E.K.)

**Keywords:** dietary supplements, cognition, cognitive performance enhancement, military, healthy young adults

## Abstract

Intake of dietary supplements has increased, despite evidence that some of these have adverse side effects and uncertainty about their effectiveness. This systematic review examined the evidence for the cognitive benefits of a wide range of dietary supplements in healthy young adult samples; the aim was to identify if any might be useful for optimising cognitive performance during deployment in military personnel. Searches were conducted in 9 databases and 13 grey literature repositories for relevant studies published between January 2000 and June 2017. Eligible studies recruited healthy young adults (18–35 years), administered a legal dietary supplement, included a comparison control group, and assessed cognitive outcome(s). Thirty-seven of 394 identified studies met inclusion criteria and were included for synthesis. Most research was deemed of low quality (72.97%; SIGN50 guidelines), highlighting the need for sound empirical research in this area. Nonetheless, we suggest that tyrosine or caffeine could be used in healthy young adults in a military context to enhance cognitive performance when personnel are sleep-deprived. Caffeine also has the potential benefit of improving vigilance and attention during sustained operations offering little opportunity for sleep. Inconsistent findings and methodological limitations preclude firm recommendations about the use of other specific dietary supplements.

## 1. Introduction

A dietary supplement is broadly defined as “a food, food component, nutrient, or non-food compound that is purposefully ingested in addition to the habitually-consumed diet with the aim of achieving a specific health and/or performance benefit” [1]. More specifically, dietary supplements include multivitamins/minerals, individual vitamins/minerals, protein and amino acids, purported prohormones, herbal (plant derived) substances, joint health products, combination products and non-categorical dietary supplements (plant, animal and synthetic derived substances) [2,3]. These may be consumed by mouth as powders, liquids, capsules or tablets. The dietary supplements market has been estimated at 132.8 billion USD in 2016, with projections this will increase to 220.3 billion USD in 2022 [4].

The use of dietary supplements is generally self-prescribed and easily accessible, with consumption increasing, particularly among healthy young adults. Consumption rates of dietary supplements in an Australian university population were found to be approximately 70% [5], with similar but lower rates identified in military populations [6,7]. Interestingly, a recent report of Australian military personnel observed an increase in the use of supplements, where 76% of men and 87% of women reported using a dietary supplement for a health benefit, indicating that usage within the Australian Army is high [8].

The observed overall increase in the use of dietary supplements within the community is alarming and has occurred despite little empirical evidence for their effectiveness and the observation of adverse effects [2,9,10,11,12,13,14,15,16,17,18,19,20,21]. Such adverse effects include insomnia (ginseng) [22], liver damage (unspecified supplements) [23], an increased risk of bleeding (gingko biloba, fish oil) [19,22], interactions with ibuprofen (gingko biloba) [24] and death (caffeine and energy supplements, respectively) [25,26]. Of particular concern is a report stating that an increasing number of dietary supplements contain unlisted potentially life threatening ingredients [27]. Nonetheless, some studies do suggest that dietary supplements may modulate some aspects of cognitive performance in healthy young adults. Supplements that have shown some cognitive benefits result from omega-3 [28,29], multivitamins and minerals [30,31] and caffeine [32].

Concurrently, defence organisations recognise the need to prepare or enhance the cognitive performance of their soldiers whilst in complex or uncertain operational environments. These commonly encompass increased exposure to a variety of stressors including sleep deprivation, climatic extremes, inadequate nutrition, physiological exhaustion and cognitive demands. Decrements in cognitive performance as a result of these stressors can be costly to the individual and the unit. For example, intense field training can increase response time by 20 milliseconds [33], which could be significant in a fire fight. Likewise, sleep deprivation can increase reaction time and errors [32], as well as negatively affect moral judgment [34] and emotional response [35]. As mistakes can be costly, it is important to identify evidence-based means of preserving or enhancing the cognitive performance of military personnel. The effectiveness, durability, and acceptability of other methods, such as computer-based cognitive training and mindfulness training are still being investigated. These other methods are also time intensive and require practice to prevent skill fade. Scientifically supported dietary supplements may provide an alternative to other methods that might be used. Given the widespread use of such supplements by healthy young adults, including military personnel, appropriate ingestion of these is likely to be more acceptable than other means of cognitive enhancement. 

The high consumption of dietary supplements by the military, the equivocal empirical evidence for a positive effect on cognitive performance, and a desire to maintain or improve cognitive performance during deployment warrant further investigation into the relationship between legal dietary supplements and cognitive performance in healthy young adults.

This literature review was undertaken in response to a request from the Australian Army for evidenced-based information on the effects of dietary supplementation on cognitive performance. Accordingly, its overall aim was to identify whether legal dietary supplements may enable a military cohort to achieve and maintain optimal cognitive performance during deployment, and which specific aspects of cognitive function can be enhanced. Thus, the focus on a ‘military context’ and how the Army could implement information was imperative and as such has been discussed throughout with this specific focus.

## 2. Materials and Methods 

Methods employed in this review followed the Preferred Reporting Items for Systematic Reviews and Meta-Analyses (PRISMA) guidelines [36]. The review protocol was registered with the International Prospective Register of Systematic Review (PROSPERO) on 19 April 2017 and was last updated 9 September 2017 (registration number CRD42017060300). The protocol modification was to (a) remove an erroneous reference to a clinical population; (b) remove a reference to a detailed analysis of the current status of supplement use and the health status of Australian Defence Force personnel, as another large study was commenced within Defence Science and Technology addressing these questions; and (c) extend the scope of keywords describing the review. The review was conducted by subject matter experts with sufficient expertise to properly evaluate the efficacy of various dietary supplements on cognitive performance. The Participants, Interventions, Comparisons, Outcomes, Study Design (PICOS) method was used to define the scope of the review (see Table 1). Papers were evaluated for beneficial outcomes in terms of one or more aspects of cognitive performance. Any adverse effects were noted to allow consideration of cost-benefits of potentially unsafe supplements. 

### 2.1. Search Strategy

The search terms used to search databases and relevant repositories were: military personnel, soldier, sailor, airmen, marine, armed forces, coast guard, submariners, army, navy, air force, combined with nutrition, dietary supplements, Dietary supplement (DS), vitamin, mineral, amino acid, protein, herb, herbal, sport drink, sport bar, nutriceuticals, food supplements, ergogenic aids, nutraceuticals, nootropics, pharmaceuticals, performance enhancement, cognitive enhancement, cognition, attention, memory, military, special forces, elite military, operations, nutritional armour/armor, and deployed. Searches were conducted of the following databases: Medline, PubMed, Scopus, Web of Science, PsycINFO, Cochrane Database of Systematic Reviews, Ovid, PsycArticles, and Science Direct. The search also included report repositories, in particular National Technical Information Service (NTIS), Defense Technical Information Centre (DTIC), RAND, National Academies Press, USAIREM, TNO, dSTL, NATO, Defence Science & Technology, Swedish Defence, Canadian Defence, French Defence and Google Scholar. Publication status was not a limitation; however, searches were limited to papers published or prepared during the period 2000–2017, with the final search completed in June 2017. The year 2000 was chosen as the early start point, because: (1) studies investigating supplement use within the military cohort were conducted after that date; (2) systematic literature reviews on some single supplements were published after that date and, with the exception of one on caffeine that covered the period 1998 onwards, most of those reviews searched databases from their inception, going back as early as the 1950s; and (3) the growth in consumption of dietary supplements is relatively new. Full search terms and key words are available in the online supplementary information. Due to the small number of papers identified, a second search was conducted including the following terms: macronutrients, micronutrients, calcium, magnesium, potassium, omega-3, omega-6, Vitamin D, Vitamin B, folate, probiotics, gut-brain axis, caffeine, flavonoids, vitamins, gingko biloba, bacopa, curcumin, ginseng, protein and tyrosine (see online Supplementary material Table S1 for a full example of the search strategy).

### 2.2. Inclusion Screening

Initially, two authors conducted independent searches of the databases. All authors were involved in the secondary search. Outcomes of both searches were collated. One author (AW) screened all identified records for relevance according to the PICOS elements. Reasons for rejection of all full-text articles were recorded. The other authors independently conducted random checks on the screening process and rationale for rejection. 

### 2.3. Data Extraction and Synthesis

At least two authors were involved during the stages of data extraction and synthesis. The abstracts of all records identified in the searches were assessed for relevance and exclusion criteria. Records were excluded if: participants were outside the age range of 18–35 years (unless there was a comparison group within this age range); there were no cognitive outcome measures; apart from multivitamin and guarana papers, a combination of dietary supplements was used (unless there was a pure supplement group to compare to placebo); and the study used a clinical population. Where the abstract provided incomplete information on these exclusion criteria the method section was read to determine if the paper should be excluded. Empirical papers passing this initial exclusion screen were read in full and included in the review unless a reason for exclusion was found on reading the paper (e.g., poor study design, confounds). Review and meta-analytical papers passing the initial screen were assessed for relevant papers fitting the inclusion criteria, and supplemented by papers produced subsequent to the original review or meta-analysis. 

### 2.4. Quality Assessment

Two researchers independently evaluated the risk of bias and methodological quality of included papers. The Grading of Recommendations, Assessment, Development and Evaluation (GRADE) [37] approach was used to assess risk of bias of all relevant papers based on these bias categories: selection, performance, attrition and reporting. Papers were judged to have a high risk of bias if they failed to use adequate randomisation methods, such as a coin toss, failed to conceal allocation, and it was unclear if blinding occurred. Overall, the risk of bias was high in 8.11% and unclear in 62.16% of papers included in this review (see Table 2). Methodological quality for all included papers including previous review or meta-analytical papers was assessed by the Scottish Intercollegiate Guidelines Network (SIGN) 50 Checklist [38]. As can be seen from Table 2, very few of the included papers were of high quality (10.81%), with the majority of low or very low quality (70.27%).

The purpose of our systematic literature review was to evaluate the potential utility of any known dietary supplements that have demonstrated efficacy for enhancing cognitive performance, and broadly, which overarching cognitive functions might be enhanced. A formal meta-analysis was not appropriate in this case due to the high number of poor quality studies. Reasons for a low-quality ranking included: an unknown risk of bias, and the heterogeneity in methodologies, and cognitive outcome measures across the included studies. Accordingly, a meta-analysis was not performed as would produce misleading results [75,76]. Studies are therefore grouped according to supplement used with a narrative synthesis of findings. However, to assist the formulation of recommendations, the total number of outcomes across all included studies and participants involved was calculated for each cognitive domain. In so doing, recommendations adopting the GRADE approach were based on the consistency of outcome findings. Outcomes could be an improvement, deterioration, or no change in cognitive performance measures. All records were documented, and all included papers were summarised according to author, design, objectives, sample, supplement information, confounds measured, outcome measures, results, and adverse effects (see Table 3; a full version of the table of evidence of included studies is provided in Table S2 of the online Supplementary material). A record was kept of excluded papers and their reason for exclusion (See Table S3 in the online Supplementary material). The PRISMA checklist was completed once the review was complete (Table S4 of the online Supplementary material) and the risk of bias was documented for all included papers. 

## 3. Results

### 3.1. Literature Search

**Figure 1:**
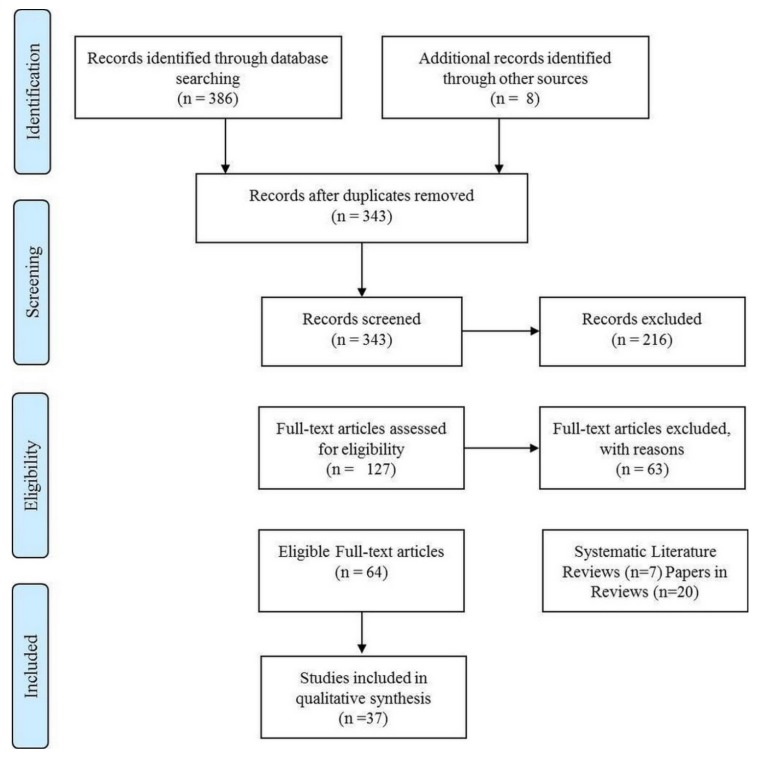
PRISMA 2009 Flow Diagram. Adapted from: Moher D., Liberati A., Tetzlaff J., Altman D.G., The PRISMA Group (2009). *Preferred Reporting Items for Systematic Reviews and Meta-Analyses: The PRISMA Statement*. PLoS Med 6(7): e1000097. doi:10.1371/journal.pmed1000097. For more information, visit www.prisma-statement.org/.

The literature search was conducted according to the PRISMA statement. The flow of information through the different phases of the systematic review is shown in Figure 1. Varied cognitive domains were assessed (see Table 4), and included psychomotor, information processing speed, attention/vigilance, memory, and executive function, using an assortment of established cognitive tasks. The search and inclusion criteria resulted in the inclusion of papers investigating the following dietary supplements: macronutrients–carbohydrates, proteins (beta-alanine, tyrosine), fats (omega-3); micronutrients–B vitamins, multivitamins, nitrate; herb (plant)-based caffeine, flavonoids, guarana, gingko biloba, ginseng; and prebiotics. Papers investigating the following supplements were excluded due to not assessing cognitive outcomes, participant age, or use of non-human participants: protein, ampakine, bacopa, curcumin, iron, nigella sativa, polyphenol, tryptophan, vitamin D, vitamin E, and zinc. The literature search also found no relevant papers for the following supplements: biotin, calcium, choline, chromium, copper, fluoride, iodine, iron, magnesium, manganese, molybdenum, niacin, pantothenic acid, phosphorus, potassium, selenium, sodium, vitamin A, vitamin C and vitamin K. Across studies, adverse effects of supplementation were generally not assessed (guarana), not reported (beta-alanine), or not observed (vitamin B, nitrate, caffeine, flavonoids, prebiotics). Only one study on beta-alanine supplementation reported physical side effects (i.e., paraesthesia (tingling) in four participants, which was due to inappropriate administration of the supplement) [39]. 

### 3.2. Overview of Cognitive Effects by Supplement

#### 3.2.1. Macronutrients

The primary macronutrients are carbohydrates, proteins and lipids, and they are required by humans to maintain health and energy. They are termed macronutrients due to the relative large amounts required (i.e., grams) for a normal diet, and are found in fruit, dairy foods, vegetables, pulses, grains, fish, and meat. Very few studies met our search criteria. The only papers that met our criteria with respect to proteins related to two of the amino acids, specifically beta-alanine and tyrosine. The only papers that met our criteria with respect to fats related to omega-3. 

##### Carbohydrates

Hoyland, Lawton and Dye (2008) [77] noted in their systematic literature review that the majority of previous reviews assessing the relationship between macronutrients and cognitive performance focused on carbohydrates, with very few studies looking at the effects of proteins or lipids. In their review they focused on identifying measures that were used to assess the impact of carbohydrate, protein, and/or lipid manipulations on cognitive function. They found 31 studies involving 1367 participants that examined acute effects of macronutrient manipulations (most notably glucose) on a range of cognitive outcome measures, including psychomotor, information processing speed, attention/vigilance, executive function, and, in particular, memory. The most consistent beneficial effects were found for memory, particularly from glucose on short-term memory, delayed memory and non-verbal memory. Nonetheless, several studies found no impact of carbohydrate supplementation on memory performance. Findings for other cognitive domains tended to be mixed, likely as a result of insufficient studies that examined these domains, and/or the use of different macronutrient manipulation across studies. More generally, beneficial effects of macronutrients tended to be more commonly observed under conditions of greater cognitive task demands. Our search failed to identify additional studies published since 2008 examining the relationship between macronutrient intake and cognitive performance in healthy young adults.

##### Beta-Alanine (Protein)

Beta-alanine is an amino acid produced naturally in the body that can also be acquired via the diet from meat and/or dietary supplements [78]. Due to its presence in other tissues, such as the brain, it is postulated that the beta-alanine precursor, carnosine, may have potential cognitive effects. Early animal studies indicate there may be a link to focus, alertness and cognitive function during stress and fatigue [79].

Only one known study to date has investigated the effects of beta-alanine (see Table 3) on cognitive performance in healthy young humans [39], where both physical and cognitive performance were assessed after four weeks of 6 g beta-alanine supplementation in a sample of military personnel. Twenty male soldiers were assessed on a variety of military tasks and a working memory task (serial subtraction) after induced fatigue from military training. Beta-alanine supplementation did not enhance working memory performance, but significant improvements in marksmanship and reaction time were found within the military context of operational task performance. GRADE and SIGN 50 evaluations of this single study employing beta-alanine was deemed low quality (See Table 2). 

##### Tyrosine (Protein)

Tyrosine is a non-essential amino acid that is used by cells to synthesise proteins. Briefly, tyrosine is found in high-protein food sources and is synthesised from phenylalanine [80]. Most importantly, tyrosine is the precursor for catecholamine synthesis, which occurs mainly in the brain or adrenal medulla. The catecholamines dopamine and noradrenaline are recognised as modulators of executive function, and are also released in response to stress. An appropriate level of dopamine and noradrenaline facilitates effective cognitive performance whereas an over- or under-abundance of either of these neurotransmitters can have an adverse effect. “Central fatigue hypothesis” [81] is a relevant military problem, where prolonged exercise and/or exposure to extreme stress (extreme temperatures, etc.) alter the synthesis and level of catecholamines. Taken together, this provides good evidence to support that tyrosine, delivered appropriately and at the right dose, can evoke positive cognitive enhancement effects. Indeed, a recent review [82] identified that tyrosine mitigated the impact of stressors such as sleep deprivation, noise, extreme climates and military combat training on a range of cognitive processes including memory, perceptual motor skills, and logical reasoning. The poor quality of papers, as assessed by SIGN criteria, and lack of physiological measures related to uptake of tyrosine meant they were unable to make firm recommendations about tyrosine supplementation; nonetheless they concluded that tyrosine may enhance cognitive performance and warranted further investigation. The current review focuses on papers published since 2000 that are not included in the Attipoe et al. [82] review.

A further eight studies (see Table 3), involving 160 participants and investigating the effect of tyrosine on cognitive processing were found to fit the inclusion criteria. Apart from one study using a single-blind crossover design [72], all studies employed a double-blind placebo-controlled crossover design to examine the role of tyrosine in mitigating stress-induced cognitive declines. Mainly female participants were recruited across the eight studies: three recruited male participants, three used female participants, and two had a mix of both sexes. Tyrosine was administered using different methods: powders, capsules and bars; and at different doses from 2 g to a total dose of 150 mg/kg (approximately 12 g for 80 kg participant) as single or double doses. Tyrosine supplementation has been demonstrated to be most effective in situations of significant stress, physical, cognitive or otherwise. As such, the studies covered: cognitive demand [66,67,68,71] and external stressors such as noise and thermal changes [69,70,72]. One study evaluated whether tyrosine would modulate the effect of transcranial direct current stimulation on working memory performance [73].

Under cognitively stressed conditions tyrosine improved working memory [66] and various aspects of executive function, namely inhibitory control [67], cognitive flexibility (smaller switching costs) [71] and creative convergent thinking [68]. Although tyrosine had no impact on cognitive performance in warm/hot environments [72], when heat stress was combined with physical exertion, tyrosine supplementation enhanced vigilance [69]. Changes in the event-related potentials N100, P300 and Contingent Negative Variation (CNV), also reflected enhanced attention and executive function related to stimulus evaluation and decision-making [70]. Tyrosine also improved working memory in unstressed individuals [73].

SIGN 50 and GRADE evaluations (see Table 2) revealed that detail pertaining to allocation concealment and appropriate blinding methodology was consistently missing amongst the included studies, resulting in the quality of the research being downgraded. However, the general finding is that tyrosine supplementation might be useful for improving some cognitive functions, specifically psychomotor skills and memory, when individuals are experiencing cumulative stress such as would occur during extended military operations with little opportunity for sleep. Although there are promising indications that tyrosine might be advantageous for improving other aspects of cognition in both stressful and stress-free environments, SIGN 50 and GRADE evaluations (see Table 2) highlight that there is insufficient quality research at this stage to reach firm conclusions or recommendations. 

##### Omega-3 (Fats)

Omega-3 is a polyunsaturated fatty acid that must be obtained through dietary intake as it is not produced naturally in the human body [83]. Eicosapentaenoic acid (EPA) and docosahexaenoic acid (DHA) are essential fatty acids present in omega-3 [84]; DHA makes up 97% of the brain’s total omega-3 fatty acid content [84], particularly in brain regions involved in attention and memory [85]. Omega-3 is important for normal cognitive development in early life and may be associated with a reduced decline in cognitive function in older adults [86]. A large proportion of the population fails to consume sufficient amounts of omega-3 [87], in particular the long chain omega-3 fatty acids found in fatty fish such as salmon, tuna or sardines, or via supplementation.

Military personnel work in conditions where maintaining optimal cognitive functioning is critical. Evidence that omega-3 can enhance cognitive performance would support moves to incorporate a greater amount of omega-3 (via food components or supplements) into operational ration packs [83]. Despite evidence that omega-3 supplementation enhances cognitive development during childhood, a recent systematic literature review found no evidence for omega-3 enhancing cognitive performance in healthy adults [88]. The current review focuses on research papers published since 2014, which was the most recent paper included in that earlier review [88].

Two further studies (see Table 3) that met inclusion criteria were identified [28,64]. In both studies participants were drawn from normal, well-rested populations, who either did not use omega-3 supplements or restrained from taking them for four weeks prior to testing. Overall 85 participants received omega-3 supplementation in these studies (54 female, 31 male). Although fish oil capsules were administered in both studies, one used an EPA-rich fish oil [64] (1680 mg EPA:1120 mg DHA per day for 35 days); and the other compared EPA-rich and DHA-rich fish oil [28] (590 mg EPA:137 mg DHA or 159 mg EPA:417 mg DHA per day for 30 days). One study assessed omega-3 uptake via changes in plasma phospholipids [28] and assessed performance on an executive function (Stroop) and spatial working memory task while recording brain activation [28]. The other study evaluated attentional control and emotional regulation [64]. 

Omega-3 supplementation had no effect on attentional control [64]. Supplementation with a high EPA:DHA ratio (3:1) for 30 days improved the selective attention and cognitive flexibility aspects of executive function as assessed with the Stroop task: reaction time decreased and fMRI patterns of brain activation indicative of increased cognitive efficiency were observed [28]. This contrasts with previous findings by Karr, Grindstaff and Alexander [89] (reported by Teo et al. [88]) that supplementation with a smaller ratio of EPA:DHA (720 mg:480 mg) had no impact on Stroop performance.

Regarding SIGN 50 and GRADE evaluations overall for the additional two papers reviewed applying an omega-3 intervention (see Table 2), the risk of bias and research quality was mixed. On the whole, although some studies have found improvements in some cognitive functions, there is little firm evidence to suggest that omega-3 supplementation can reliably enhance cognition in healthy young adults. More research is required in the future to provide a recommendation. 

#### 3.2.2. Micronutrients

Micronutrients are essential elements required in small quantities, but necessary to remain healthy. They include vitamins and minerals. Very few papers met our search criteria. 

##### B Vitamins

The B vitamins are a group of water soluble vitamins comprising thiamine (B_1_), niacin (B_3_), pantothenic acid (B_5_), vitamin B_6_, biotin (B_7_), folate (B_9_), and vitamin B_12_. The B vitamins collectively play a critical role at all levels of brain function as co-enzymes and precursors of enzymatic processes. They are important for energy production, DNA/RNA synthesis/repair, genomic/non-genomic methylation, and the production of neurochemicals and signalling molecules [30,90]. The mechanisms by which B vitamins affect cognition are still unknown. The B vitamins folate, B_6_, and B_12_ are known to be involved in the modulation of homocysteine levels; this is important as elevated plasma homocysteine has been associated with poor cognition. Although there is no firm evidence of this relationship, it is likely that the association of the B vitamins with homocysteine metabolism is a by-product of other unknown biological factors that impact on cognitive function [91].

There is a shortage of well-controlled studies assessing the impact of supplementation of B vitamins on healthy young adults (see Table 3). One study assessed the effect of folate, vitamin B_6_, and vitamin B_12_ supplementation [74] on cognitive function in 56 healthy young females with no external stressors. High doses of B vitamins (750 µg folate, 15 µg vitamin B_12_, 75 mg vitamin B_6_) were administered using tablets or capsules, for five weeks [74]. Information processing speed, attention, memory and executive function were measured. There was a trend towards supplementation with folate, vitamin B_6_ and vitamin B_12_ enhancing aspects of memory performance. There was no impact on other cognitive measures. GRADE and SIGN 50 evaluations (see Table 2) revealed this study to be of low quality with a high level of bias risk. More research is required to determine the effect of the vitamin Bs on cognitive performance. 

##### Nitrate

Nitrate is obtained in the diet through the consumption of nitrate-rich vegetables, including beetroot, broccoli, lettuce, and spinach [63]. Although there is limited research on the effects of nitrate supplementation on cognitive functions in humans, the beneficial effect of nitrate on cognition appears to be related to its conversion to nitric oxide. Nitric oxide is involved in the modulation of cerebral blood flow, which is important for optimal brain function. In older adults, dietary nitrate supplementation resulted in improved cerebral blood flow to areas of the brain related to executive functioning [92].

Two studies involving 56 participants have assessed the effect of a dietary nitrate supplementation on cognitive performance in healthy young adults (see Table 3). A double-blind crossover design [62] and a double-blind, placebo-controlled, parallel groups design [63] were employed. Participants were non-military and were either recreationally active men [62] or a mixed male and female sample [63]. Nitrate supplementation was assessed under different conditions: during a physically demanding task (cycling) [62] and in response to a cognitive demand battery [63] to measure cognition. Both studies assessed the effect of single doses of a similar nitrate-rich drink (beetroot juice with added apple and blackcurrant juice; containing 5 to 5.5 mmol of nitrate). Performance on measures of attention (rapid visual information processing test, RVIP) [62,63], working memory (serial subtraction) [63], and executive function (Stroop) [62] were assessed.

The beetroot juice did not enhance attentional performance [63], nor did it mitigate the deterioration in attentional abilities as a result of increasing exercise intensity [62]. Wightman et al. [63] observed greater accuracy on serial threes subtraction but not serial sevens subtraction. However, this effect should be interpreted with caution as the nitrate group underperformed at baseline. No positive effects of beetroot juice on executive functioning were observed [62]. GRADE and SIGN 50 assessments (Table 2) of research on nitrates denoted the studies to be of acceptable-high quality and had low risk of bias, indicating well designed and executed research. Although negligible improvements were found, the small number of papers investigating the impact of nitrates and cognitive performance indicate more research in this domain is warranted. 

#### 3.2.3. Herbal (Plant-Based) Supplements

Herbal supplements include plant-based extracts such as caffeine, flavonoids, Gingko biloba, ginseng and guarana. Of these, the majority of papers focused on caffeine. 

##### Caffeine

Caffeine is a plant alkaloid that is quickly absorbed and found in food and drinks such as coffee, tea, energy drinks, soft drinks, and chocolate [93]; peak caffeine concentrations are reached between 15 and 120 min after ingestion [94,95]. The energising and concentration boosting qualities of caffeine are well-known and are the reason caffeine is the most commonly used psychostimulant [41,96]. A growing body of literature has investigated the effects of caffeine on cognitive performance, particularly during/after sleep deprivation. Caffeine works by blocking adenosine receptors within the brain and has demonstrated positive changes (at varying doses) on the alerting, orienting and executive control attention networks within the brain, specifically enhancing alertness, vigilance and reaction time. It has not demonstrated improvements in memory performance or other executive functions, such as decision-making [94]. A recent systematic literature review found caffeine supplementation to be promising for maintaining or improving several aspects of cognitive performance in sleep-deprived people. These are tasks requiring attention, executive function and information processing speed [97]. The current review extends that of Crawford et al. [97] by including studies they omitted and/or were published after 2014.

A further seven studies (see Table 3) were found that examined the effect of caffeine on cognition in healthy young adults. Four studies used crossover designs and three utilised independent group designs. All studies considered acute effects of caffeine and had a relatively even distribution of male and female participants. Two studies recruited men only [43,44] and one recruited military personnel [44], with a combined total number of 172 participants. Participants’ caffeine consumption was heterogeneous across all included studies, examining low, moderate, high and mixed users. Caffeine was commonly dosed at 200 mg (range 50–400 mg), with a maximum daily administration of 800 mg. A range of administration modes was used, including capsules, chewing gum, prepared beverage (decaffeinated coffee and caffeinated coffee), or dissolved in distilled water. Four studies examined caffeine in the context of sleep deprivation, or compared well-rested and sleep-deprived subjects [40,44,45]. (The Aidman et al. (2018) [40] paper was under review at the time of conducting the literature search for this systematic review. It has since been published.) The assessed areas of cognition were: psychomotor, information processing speed, attention/vigilance, memory, executive function, and a combination of cognitive abilities required for a practical test of simulated driving ability.

In sleep-deprived participants, caffeine enhanced the performance of military personnel on attentional tests [44]. Further, repeated doses of caffeine mitigated the decline in information processing speed, vigilance, and logical reasoning associated with sleep deprivation [44]. Similar results have been found during a driving task undertaken by sleep-deprived [40,45] and sleep-restricted (Study 1) [45] university students. Driving a vehicle requires attentional control functions of alertness, orienting and executive attention, all of which can be impaired by sleep deprivation. Despite one of the driving studies measuring drowsiness objectively with the Johns Drowsiness Scale [40], and the other measuring subjective sleepiness [45], both studies found that drowsiness/sleepiness after sleep deprivation impaired driving ability. Caffeine mitigated this effect, and subsequently, driving ability did not deteriorate to the same extent after caffeine was consumed. Caffeine also mitigated the effect of sleepiness on driving ability after restricted sleep (Study 1) [45]. Similar improvements in executive control performance were found in sleep-deprived and well-rested participants, with caffeine ameliorating the effect of sleep deprivation on logical reasoning [44].

In well-rested individuals, caffeine improved memory (recognition) and aspects of information speed, specifically, choice reaction time but not simple reaction time [43]. In addition, 400 mg of caffeine enhanced executive function (conflict resolution) and alertness, but not selective attention (as measured by the Attention Network Task–ANT) [41]. Furthermore, although one standard cup of caffeinated coffee improved executive function when measured by the ecologically valid Jansari Assessment of Executive Function (JEF), it failed to do so when assessed by the Stroop task [46]. This suggests that one standard cup of coffee does not influence executive function, or that traditional tests of executive function such as the Stroop task may lack the sensitivity to detect the enhancing effects of caffeine. Caffeine was found to have no effect on other aspects of memory performance in well-rested participants [42].

Taken together, findings from the well-controlled studies suggest that the appropriate dose of caffeine might enhance attention, memory, problem solving and logical reasoning in sleep-deprived young adults. Further, this supplement might also be used to mitigate the joint effect of sustained operations and sleep deprivation on attention and vigilance. Further quality research is necessary before definitive conclusions can be reached about other cognitive functions or contexts. 

##### Flavonoids

Flavonoids are natural polyphenol compounds found in fruits and other plants such as berries, apples, wine, tea, and cocoa [48]. Sub-classes of flavonoids include isoflavones (in soybeans and peanuts), flavanols (in tea and cocoa), flavonols (in fruits and vegetables), flavones (in cereals and herbs), anthocyanidins (in berries), and flavanones (in citrus fruits) [98]. The actions of dietary flavonoids on cognition appear to be related to various potential actions on the brain, including neuroprotection from neurotoxins and neuro-inflammation, synaptic signalling activation and improved cerebrovascular blood flow. These actions are driven by the apparent ability of flavonoids to interact with neuronal signalling cascades in the brain, resulting in the inhibition of cell death via exposure to neurotoxic species, the promotion of neuronal survival and differentiation, and an enhancement of peripheral and cerebral blood perfusion. Effects of flavonoids on cognition are likely the result of optimal maintenance of brain morphology due to the regulation of neuronal signalling and protection against neuronal losses [99].

Four included studies considered acute effects of flavonoids on cognitive performance in a total of 137 young healthy adults. All studies utilised crossover designs and were conducted in non-military populations. Although each study used participants from both genders, there was a greater proportion of female participants. The sub-classes of flavonoids investigated were flavanones, cocoa flavanols, anthocyanins, and epigallocatechin gallate (EGCG; a flavonoid typically found in green tea). The flavonoid dose and administration methods were as follows: 70.5 mg flavonones in a commercial citrus juice [47], 525 ± 5 mg of polyphenols per 60 kg of body weight in a blackcurrant extract and a blackcurrant fruit juice, [49] 520 mg and 994 mg of cocoa flavanols in a dairy-based cocoa drink [48], and 135 mg and 270 mg of EGCG in capsules [50]. All studies investigated the effects of single doses of flavonoids. Information processing speed, attention/vigilance, memory, and executive function were measured. Two studies used cognitive test batteries intended to increase the cognitive demand placed on participants [48,49].

Flavonone supplementation in citrus juice improved information processing speed (digit symbol substitution task) but not inhibitory control (Go/No-Go task) [47,49]. Blackcurrant extract mitigated deteriorating accuracy in sustained attention (RVIP). In addition, Scholey et al. [48] found that the optimal dose of cocoa flavanols (520 mg) improved working memory, in terms of serial threes subtraction, but not serial sevens. Conversely, Wightman et al. [50] found no effect of EGCG on working memory (either serial threes or serial sevens subtraction). Across all studies, flavonoids had no impact on executive function [47,50]. Despite the high quality of two of the included papers (see Table 2), there still remains insufficient quality empirical support to allow us to make any recommendations regarding flavonoid supplementation. 

##### Gingko biloba

Gingko biloba is an herbal supplement derived from extracts in the leaves of the gingko biloba tree [52] and is commonly used in a standardised form in clinical studies [100]. Unless otherwise specified, the studies included in this review administered a standardised gingko biloba extract. The active molecules of gingko biloba are believed to be linked to an array of potential physiological effects which can influence cognition. These physiological effects include [54]: the scavenging and inhibition of free radicals, anti-platelet activating factor, reducing neuronal death, improved blood circulation, increased cerebral perfusion, and protection against hypoxia. Gingko biloba has been claimed to improve short-term memory, rate of learning, and reaction time [52].

Six articles (comprising seven studies) met inclusion criteria and examined the effect of gingko biloba supplementation on cognition in healthy young non-military adults (see Table 3). There were three male-only studies and four that comprised predominantly female participants. In total, the impact of gingko biloba on cognitive performance was assessed in 240 participants (126 male; 114 female). Crossover (four) and independent group (three) designs were employed. Generally, a single administration of gingko biloba (tablet or capsule: 120–360 mg) was examined with the exception of two studies which applied extended supplementation for six weeks [51] or five days [55]. Kennedy et al. [54] also administered two gingko biloba extracts complexed with phospholipids in addition to the commonly used gingko biloba extract.

Apart from the vigilance component, neither gingko biloba nor ginkgo biloba complexed with phospholipids consistently improved speed of attention [53,54]. A dose-dependent effect of gingko biloba was found with 240 mg and 360 mg improving speed of attention at 2.5 and 6.5 hours post dosing [52]. Reaction time in a sustained attention task was improved after a single dose of 120 mg of gingko biloba; however, this effect may have been due to differences between the intervention and placebo conditions at baseline [51].

All studies assessed the effect of gingko biloba on memory; whilst improvements were found, this was not consistent across all memory tasks; dose-dependent effects were observed for gingko biloba, and in some cases differential effects were found for gingko biloba and ginkgo biloba complexed with phospholipids. A 360 mg dose of gingko biloba enhanced speed of memory, whereas a 240 mg dose degraded performance [52]. A 120 mg dose of ginkgo biloba or 120 mg gingko biloba complexed with phosphatidycholine generally had no impact on overall speed of memory [52,53,54]. Conversely, gingko biloba complexed with phosphatidylserine enhanced overall speed of memory, as well as memory accuracy in picture recognition [54]. Longer durations of gingko biloba supplementation (six weeks [51] or five days [55] also failed to enhance memory ability. Working memory (serial sevens subtraction speed) was enhanced four to six hours post-dose [53]; however, this is in contrast to Scholey and Kennedy [56], who observed improved accuracy. A single 120 mg dose of gingko biloba improved secondary memory, a composite score derived from performance accuracy on delayed word/picture recognition, and immediate/delayed word recall tasks, with this effect being maximal one hour post supplementation [54]. Nil or minimal effects were observed for: reaction time [55], attention [51], working memory (including serial threes subtraction) [53,54,55], and executive function (mental flexibility or planning ability) [51].

In addition, extended supplementation of gingko biloba (>1 week) failed to elicit any cognitive improvement [51]. It should be noted that Kennedy et al.’s studies [52,53,54] combined results from individual tests into the cognitive factors of speed of attention, accuracy of attention, speed of memory and quality of memory, all derived from a factor analysis of the Cognitive Drug Research computerised assessment battery. However, in some of their studies they evaluated performance on underlying tasks when there was no impact on the overall cognitive factor [54].

In summary, the studies investigating the impact of gingko biloba on cognitive performance yielded mixed results. This fact, together with the uncertainty about risk of bias and poor paper quality (see Table 2), prevents us from making any recommendations about the use of gingko biloba as a means of enhancing cognitive performance. Nonetheless, further quality research is warranted that examines in more detail the dose- and time-dependent effects. 

##### Ginseng

The plant Panax ginseng, commonly known as ginseng, is a traditional Chinese treatment and herbal supplement derived from ginseng roots [53]. The major active molecules of Panax ginseng are ginsenosides (saponins), comprising of 30 identified types [101]. Currently, the mechanisms that explain the cognitive effects of ginseng are not known [102]. Ginsenosides have been demonstrated to cause many forms of physiological effects, including modulation of the cardiovascular immune response systems, deceleration of platelet aggregation, modulation of neurotransmission, and nitric oxide synthesis [102].

A recent literature review identified that the included controlled studies consistently found that ginseng enhances cognitive performance, particularly aspects of working memory and speed of attentional processing [103]. Indeed, this review identified that ginseng was as effective, if not moreso, at enhancing working memory and reaction time than was the pharmaceutical modafinil, which is commonly used to enhance cognitive performance in sleep-deprived individuals and more recently in rested people. Nonetheless, Neale et al. [103] also identified that there is limited evidence available to form strong conclusions on the efficacy of ginseng for cognitive enhancement. An earlier systematic literature review reached similar conclusions: empirical studies have identified a potential benefit of ginseng for cognitive enhancement; however, there are too few randomly controlled crossover designed studies to enable strong recommendations on the use of ginseng for cognitive enhancement—or the durability of such enhancement [104]. The current review extends that of Geng et al. [104] and Neale et al. [103] by including studies they omitted and/or were published after 2011.

One additional study (see Table 3), subsequent to those reviewed in earlier systematic reviews, was found that assessed the cognitive enhancing effects of ginseng supplementation in 15 healthy young adults. This study used a randomised double-blind independent groups design with a placebo control and administered Korean ginseng at a dose of 4500 mg per day for two weeks. No effects were found on vigilance; however, ginseng administration had beneficial effects on brain activity related to attention and working memory, as indexed by the P300 event-related potential [57]. SIGN and GRADE evaluations (Table 2) of this single study highlighted the need for caution in interpretation as it was found to be of high risk and very low quality. In summary, findings from the included study will not change the findings from the previously performed systematic reviews of ginseng [103,104]; limited evidence is available to support that ginseng is efficacious in enhancing cognition in healthy young adults. 

##### Guarana and Multivitamins

Guarana is a plant extract used predominantly as a food additive and is generally consumed with other herbal supplements, such as ginseng [60]. Guarana’s stimulant properties have been attributed to its caffeine content and high quantities of saponins and tannins [60]. The cognitive benefit of guarana is proposed to be related to its ability to decrease the physiological response to physical or psychological stressors [59]. It has been proposed that the impact of guarana on cognition is due to the synergistic effect of its constituents, such as caffeine [105] and theanine [106].

Four studies, involving a total of 224 participants, examined the effect of acute doses of guarana on cognition (see Table 3). Two utilised crossover designs [59,61] and two used an independent groups design [58,61]. Sample compositions varied across the studies: predominantly female (two studies), approximate even gender distribution (one study), and active males only (one study). A standardised guarana extract was used by Kennedy et al. [59] (75 mg) and Haskell et al. [58] (37.5 mg, 75 mg, 150 mg, 300 mg). The other two studies administered a multivitamin + guarana supplement (Berocca Boost®, hereby referred to as MVM + G) in the form of an effervescent tablet dissolved in water. The MVM + G contained 222.2 mg of guarana (including 40 mg caffeine), and equal to or above the recommended dietary allowance (RDA) of B vitamins. The two MVM + G studies induced fatigue using a traditional cognitive demand battery [60] or exercise in combination with a repeated battery of cognitive tests [61]. Attention, memory, working memory, and executive function were measured.

Both MVM + G studies measured attention using RVIP. While Kennedy et al. [60] found an improvement in RVIP speed and accuracy, Veasey et al. [61] did not, nor on choice reaction time. Veasey et al. suggested that this was likely due to a lower number of repetitions completed on the tasks, as compared to Kennedy et al. Alternatively, in contrast to Kennedy et al.’s traditional cognitive demand battery, Veasey et al.’s battery in combination with their exercise regime may not have induced enough mental fatigue to detect any beneficial effects of the MVM + G drink on attention.

Kennedy et al. [59] also found mixed results from pure guarana extract supplementation: speed, but not accuracy, of attention was enhanced by guarana. Speed of attention was a composite of simple reaction time, choice reaction time and digit vigilance, whereas accuracy of attention was a composite of choice reaction time and digit vigilance. In addition, secondary memory (a composite of immediate and delayed recall and recognition tasks) was improved 2.5 h post-supplementation, and at three time points specifically for picture recognition (1, 2.5 and 4 h post-supplementation).

Inconsistent results were also found for memory performance. MVM + G had no effect on working memory (serial threes and serial sevens subtraction) [60]; however, it improved accuracy on numeric working memory [61]. MVM + G did not improve word recall, word recognition, or picture recognition accuracy, but it did improve speed of picture recognition [61]. A standardised guarana extract, however, improved performance on serial sevens subtraction, a sentence verification task, and secondary memory, but had no effect on working memory, speed of memory, or serial threes subtraction performance. Apart from working memory, executive function was not assessed in the MVM + G studies. However, Kennedy et al. [59] and Haskell et al. [58] did examine one aspect of executive functioning, logical reasoning, which was not enhanced by the pure guarana extract. 

Overall SIGN and GRADE evaluations of the included studies for guarana intervention (see Table 2) identified that the risk of bias was unclear and the quality of the studies to be low. Although no recommendation can be given for the efficacy of guarana for cognitive enhancement, further research using well-controlled studies might shed more light on the dosage and time effects of this supplement on cognitive performance. 

#### 3.2.4. Prebiotics

Prebiotics are non-digestible foods/compounds that are found in a range of foods, such as asparagus, banana, garlic, onion, beans, and lentils. They are comprised of carbohydrates or short chains of saccharide molecules [107]. An important characteristic of dietary prebiotics is that they must be a selectively (i.e., preferential to gut microbiota populations including Lactobacillus and Bifidobacteria) fermentable ingredient which aids the composition and/or activity of the gut microbiota [108]. Different forms of prebiotics exist that commonly include inulin, fructo-oligo-saccharides (FOS), and galacto-oligo-saccharides (GOS). Early evidence suggests that through post-fermentation by resident gut microbiota, prebiotics are capable of modulating a central brain growth factor (BDNF) among other plasticity-related proteins, neurotransmitters, cytokines, as well as anxiety, and emotional processing [107].

Only one study to date (see Table 3) has examined the effect of prebiotics on cognition in 50 healthy young adults. Smith et al. [65] used a crossover design to assess the effect of an acute dose of oligofructose-enriched inulin on reaction time, attention, and memory. A 5 g dose of the inulin supplement was administered in the form of powder added to a decaffeinated tea or coffee which was consumed with breakfast. There was no effect of oligofructose-enriched inulin on simple reaction time or measures of attention [65]. Inulin supplementation enhanced episodic memory (free recall and delayed recognition); however, it did not improve semantic processing, spatial memory, or executive function (logical reasoning). SIGN50 and GRADE assessments (see Table 2) of this one study on prebiotics identified that it was of acceptable risk and acceptable quality. More quality research in the area of utilising a prebiotic for cognitive enhancement in healthy young adults is required for appropriate recommendations to be made. 

## 4. Discussion

### 4.1. Scope of Review

The aim of this systematic review was to investigate the relationship between legal dietary supplements and cognitive performance in healthy young adults. Specifically, we sought to determine whether the intake of any such supplements could preserve or enhance cognitive performance with a view to optimising such performance during deployment for the modern war fighter. Our review extended previous reviews in two important ways. First, it included studies of healthy young adult samples more generally, as the results have wider applicability beyond the military. Second, our review included a wide range of dietary supplements, such as various macronutrients and micronutrients, as well as biologically active non-nutrients. Studies included in the current review covered a range of cognitive areas. Individual studies generally focused on one or more of the following cognitive domains: psychomotor, information processing speed, attention/vigilance, memory, and executive function.

### 4.2. Overall Synthesis

Overall there appears to be some evidence of cognitive enhancement from dietary supplements in healthy young adults. Several studies reported beneficial effects on information processing speed, in particular from supplementation with flavonoids [47] and guarana [60]. Others showed positive effects on memory following supplementation with tyrosine [66,73], caffeine [43], flavonoids [48], gingko biloba [54,56], ginseng [57], and prebiotics [65]. In addition, enhanced effects on attention were observed in some studies that supplemented with tyrosine [69,70], caffeine [44] or ginseng [57], and a handful of studies showed improved executive function with tyrosine [67,68,71], omega-3 [28], or caffeine supplementation [43,44].

However, as shown in Table 5, these beneficial effects were not observed either across all cognitive domains or for all aspects of a particular cognitive domain. For example, Veasey et al. [61] found no effect of guarana on visual information processing speed but they did for recognition memory. In addition, the single study on prebiotics found positive effects only for episodic memory but not semantic memory [65]. Furthermore, Hoffman et al. [39] found that beta-alanine enhanced psychomotor performance, but not working memory. Likewise, flavonoids enhanced information processing speed [47], but not executive function [47,50].

For the majority of dietary supplements included in this review, findings were inconsistent across studies. While some studies showed positive effects of supplementation on psychomotor, information processing speed, attention/vigilance, memory, and/or executive function, others did not. This was notably the case for tyrosine [66,67,68,69,70,71,72,73], caffeine [40,41,42,43,44,45,46] and guarana [58,59,60,61]. By contrast, several supplements, namely omega-3 [64], B vitamins [74], and nitrates [62,63] showed very little, if any effect on cognitive performance in healthy young adults. However, in terms of military specific tasks, supplementation with beta-alanine was shown to improve marksmanship [39].

### 4.3. Situational Effects

Interestingly, several studies showed enhanced cognitive performance from supplementation under specific conditions. In particular, when macronutrient manipulations involving carbohydrates yielded beneficial effects, this occurred specifically under high cognitive task demand [77]. Furthermore, supplementation with tyrosine improved some aspects of memory and executive function following exposure to a cognitive stressor, but had no impact on the effect of a physical stressor (see Table 5) [66,67,68,69,70,71]. However, [69] did find that tyrosine improved vigilance during physical exercise in the heat. Thus, unless a certain threshold of physiological stress is reached, tyrosine by itself does not improve cognition. In addition, flavonoid supplementation produced stronger cognitive effects in the two studies that induced cognitive demand [48,49]. Although Kennedy et al. [60] observed stronger cognitive effects from guarana supplementation administered under cognitive demands, Veasey et al. [61] did not when participants also exercised; however, the cognitive load in the latter study may have been insufficient to induce mental fatigue. Likewise, a number of studies found that caffeine mitigated the effects of sleep deprivation on reaction time, recognition, vigilance and overall executive functioning, including in military samples [40,44,45]. In addition, some recent studies showed improvements in memory and executive function from caffeine consumption in well-rested individuals [43]; however, others did not [42].

### 4.4. Limitations

The studies included in the review are subject to various limitations. First, there was substantial variability in sample size and composition across studies. Sample sizes ranged from as few as 11 participants to more than 200 participants, with small sample size a frequent occurrence, yielding insufficient power to detect enhanced cognitive effects. Military samples were predominantly made up of men, whereas samples of healthy young adults typically included roughly equal gender ratios, or slightly more women.

Second, the quality, purity, ratio, duration, dose, and timing of the supplements varied widely across studies. In terms of quality, some studies did not administer the supplement in its pure form; it also contained other active ingredients, which could have been responsible for, or have contributed to, any enhanced cognitive effects. For example, the guarana extract can also naturally contain up to four times the amount of caffeine as found in coffee beans [109], with some studies also combining it with B vitamins [60,61], while the nitrate-rich beetroot juice not only contained nitrate but also flavonoids and flavonols [63]. Relatedly, different sub-classes of flavonoids were used across flavonoid supplementation studies [47,48,49,50], while omega-3 supplementation studies have been found to suffer from varied EPA to DHA ratios [88], as well as the use of oxidised supplements in some studies [110]; all of these could have contributed to the current state of mixed findings. In addition, potential cognitive effects could have been masked by (a) under-dosing, both in terms of low dosages, and (b) short supplementation periods. Doses considered to be low were used in several omega-3 [28], flavonoid [47,48,50], and ginseng [111] studies, whilst the short supplementation period in Lamport et al. [47] resulted in insufficient absorption time, as the cognitive effects of flavonone-infused citrus juice were evaluated only two hours after supplementation. Finally, although the majority of included studies did use a placebo-control group or a crossover design, the timing of supplementation (i.e., time of day of administration) was not always mentioned, and thus variations in circadian rhythms could have minimised or amplified any cognitive effects. As the gastrointestinal system and metabolism are regulated by circadian rhythms [112,113], it is important to control for the impact of the circadian system on the absorption and metabolism of the ingested supplement.

Third, studies varied in terms of the cognitive domains that were assessed, with limited or no investigation of executive function following supplementation with beta-alanine, gingko biloba, ginseng and guarana. Relatedly, different tasks were used to measure a particular cognitive function across studies.

Fourth, study designs did not consistently control for practice effects (ginseng) [57] or pre-existing baseline differences between the supplementation and placebo groups (gingko biloba) [51], or did not include a placebo-control group altogether (omega-3) [28].

Finally, SIGN50 scoring identified a substantial number of included papers of low quality (see Table 2). To make firm recommendations about the use of dietary supplements for cognitive enhancement, it is crucial that researchers are aware of the need to provide full information on their methodology, especially randomization and blinding techniques, to ensure papers are of high quality and reduce liability of risk and bias. Teo et al. [88] made a similar recommendation in their review on the effect of omega-3 on cognition and highlighted the importance of researchers following agreed reporting guidelines.

These methodological limitations could account for some of the conflicting results regarding cognitive effects from dietary supplementation. Methodological variations among studies also make it difficult to directly compare them, and thus draw firm conclusions. Moreover, the risk of bias assessment deemed the risk of the majority of studies to be unclear. In addition, the quality assessment indicated considerable variability in the quality of studies, with very few studies of high quality.

Of interest, two studies on the cognitive effects of probiotic supplementation were excluded for inappropriate study design [114] and being outside of age-range [115]. Nevertheless, evidence in animals and clinical populations suggests that further research into the cognitive effects of probiotics is warranted. Finally, the cognitive effects of many dietary supplements, such as protein, iron, magnesium, trace elements (e.g., selenium), and many others have not previously been investigated in quality randomised control studies in a cohort of healthy young adults aged 18–35 years. This in itself offers potential scope for future research. 

### 4.5. Recommendations

At present there is limited high-quality research on the cognitive effects of dietary supplements in military samples, and in healthy young adults more generally. Nevertheless, the current review suggests that some legal dietary supplements could benefit the cognitive performance of healthy young adults, thus military personnel. First, based on the conditional GRADE evaluation (Table S5 in online Supplementary material), we suggest tyrosine could be used to mitigate the impact of physiological stress (particularly sleep deprivation) on psychomotor and memory performance. Second, a conditional recommendation can also be given for caffeine as it was commonly effective for mitigating the effects of sleep deprivation on attention, vigilance and aspects of executive function. Caffeine potentially also has the additional advantage of enhancing attention and vigilance when military personnel are on sustained operations, so not only working intensively for long periods but also sleep-deprived. However, the quality of caffeine studies was much more varied than that of the tyrosine studies with a mixture of acceptable, unclear and low-quality studies, as also reported in the review by Crawford et al. [97]. Further, although rare, large doses of caffeine can be fatal [25]. We therefore suggest that although caffeine could be used to reduce the impact of sleep deprivation and physiological fatigue on cognitive performance, the dosage provided should take into consideration other sources of caffeine intake. Finally, the lack of quality randomised controlled studies precludes us from offering any qualified recommendations about other supplements covered in this review. 

### 4.6. Future Directions

Future directions would encompass a three-pronged approach. First, there is a clear need for sound research into the cognitive benefits of legal dietary supplements, especially among healthy young adults, including military personnel, who increasingly take such supplements despite limited scientific evidence for their efficacy. In particular, future research should consist of adequately powered and well-conducted randomised double-blind placebo-controlled studies. Studies should use adequately dosed high-quality supplements, and determine the appropriate time of day of administration. Furthermore, studies would ideally use crossover designs with appropriate duration to allow for wash-out effects, and focus on the effect of supplements on cognitive functions that have been identified as critical for the military operational environment. With respect to military contexts, study designs should include military specific tasks and conditions that mimic operational stress, such as cognitive load and exposure to stressors, both external (e.g., climatic extremes, noise, vibration) and internal (e.g., sleep deprivation, inadequate nutrition, dehydration)). Nutritional intake both in garrison and during field/operational deployments is commonly found to be inadequate within military populations [116,117] and should therefore be considered in future analyses. To ensure findings generalise to healthy young adults more broadly, the cognitive effects of dietary supplementation should also be tested in individuals who have had adequate sleep (duration and quality), as this will also identify which, if any, supplements would be useful for enhancing cognitive performance as opposed to mitigating its decline when a person is in a stressful environment.

Second, research should address the safety considerations of any supplements that do demonstrate cognitive benefits, and thus weigh up the pros and cons of taking them. The current review identified very few adverse effects from dietary supplementation, indicating that the wide range of supplements reviewed here are generally considered to be safe. However, anecdotal evidence from military samples, and some empirical evidence [2,9,10,11,12,13,15,16,17,18,19,20,21], indicate that some dietary supplements may be harmful, and worryingly, people continue to take a supplement even if they experience bad side effects. Further, despite warnings about their potential harmful effects and the lack of regulation, a recently reported study in the United States found that 50% of people surveyed in 2004–2015 believed that dietary supplements were regulated, safe, and that manufacturers were required to provide visible information on their side effects [26]. A recent study found that the National Institutes of Health (US) Dietary Supplements Label Database identified supplements that claimed to enhance cognition but contained potentially dangerous ingredients such as vinocetine, huperzine A, and picamilon [118]. Thus, it is imperative that future studies report adverse effects to enable the creation of a database to inform regulators of the need for better control of dietary supplements.

Third, because of the potential adverse effects of dietary supplements, research should focus on developing appropriate behaviour change programs designed to equip healthy young adults with the skills and/or support structures needed to make informed safe choices on dietary supplements that they may become exposed to through marketing hype and/or word of mouth.

Having a cognitive edge will help defence personnel achieve superiority in demanding and uncertain environments. Although firm recommendations cannot be made due to a shortage of well-designed studies, conditional recommendations can be made for caffeine and tyrosine, which can enhance aspects of cognition under specific conditions. Further high-quality research is needed to ascertain whether caffeine and/or tyrosine can enhance a broader range of cognitive function more generally. Comparative studies comparing the benefits of caffeine and tyrosine are also warranted, as these would identify whether a single dietary supplement would suffice across a range of stressful and non-stressful situations. In addition, nitrates and omega-3 appear to have little benefit for cognition in healthy young adults. Nevertheless, sufficient evidence exists to warrant further research into the effects of some supplements in healthy young adults. Some critical questions that need be addressed in future research include: (1) Can tyrosine mitigate psychomotor deficits due to sleep deprivation? (2) What are the appropriate protocols for caffeine to ameliorate the detrimental effects of sleep deprivation on attention, memory, and executive function? (3) Can caffeine enhance memory and executive function in well-rested individuals? and (4) What are the appropriate EPA:DHA ratios that result in omega-3 enhancing memory and inhibitory control, and to what extent is this task-dependent?

## Figures and Tables

**Table 1:** Criteria (Participants, Interventions, Comparisons, Outcomes, Study Design–PICOS) used to define the scope of the review.

**Parameter**	**Description**
Population	Healthy young adults in both military and civilian populations aged 18–35 years to reflect the age of military personnel likely to be deployed. Reports using experimental or control groups outside the 18–35 age range were included where results for this age group could be clearly identified.
Intervention	Oral administration of legal dietary supplements, used a sole nutritional element, with the aim of enhancing cognitive performance. Multivitamin and/or multi-mineral supplements were included as they are consumed by a large number of military personnel.
Comparison	Age-matched controls with placebo or no treatment, or repeated samples designs and placebo.
Outcome	Cognitive domains: psychomotor, information processing speed, attention/vigilance, memory, and executive function.
Study design	Peer-reviewed randomised control trial

**Table 2:** Grading of Recommendations, Assessment, Development and Evaluation (GRADE) risk of bias and Scottish Intercollegiate Guidelines Network (SIGN) quality of randomised controlled trials evaluations.

	Random Sequence Generation (Selection Bias)	Allocation Concealment (Selection Bias)	Blinding of Participants and Personnel (Performance Bias)	Incomplete Outcome Data (Attrition Bias)	Selective Reporting (Reporting Bias)	Other Sources of Bias	Risk of Bias	SIGN Quality Evaluation
**Beta-Alanine**								
Hoffman 2014 [39]	**-**	**-**	**?**	**+**	**+**	**+Funding**	**-**	**?**
**Caffeine**								
Aidman 2018 [40]	**+**	**+**	**?**	**+**	**+**	**+**	**+**	**+**
Brunye 2010 [41]	**+**	**?**	**?**	**+**	**+**	**+Funding**	**?**	**-**
Hussain 2015 [42]	**-**	**-**	**?**	**+**	**+**	**+**	**-**	**?**
Kahathuduwa 2017 [43]	**+**	**+**	**?**	**+**	**+**	**+**	**+**	**+**
Kamimori 2015 [44]	**-**	**?**	**?**	**+**	**+**	**+**	**?**	**-**
Reyner 2000 [45]	**+**	**?**	**+**	**+**	**+**	**+**	**+**	**+**
Soar 2016 [46]	**+**	**?**	**+**	**+**	**+**	**+**	**+**	**+**
**Flavonoids**								
Lamport 2017 [47]	**+**	**+**	**+**	**+**	**+**	**+Funding**	**+**	**++**
Scholey 2010 [48]	**+**	**?**	**?**	**+**	**+**	**+Funding**	**?**	**-**
Watson 2015 [49]	**+**	**+**	**+**	**+**	**+**	**+Funding**	**+**	**++**
Wightman 2012 [50]	**+**	**?**	**?**	**+**	**+**	**+**	**?**	**-**
**Gingko Biloba**						**+**		
Elsabagh 2005 [51]	**-**	**?**	**+**	**+**	**+**	**+**	**?**	**-**
Kennedy 2000 [52]	**+**	**?**	**?**	**+**	**+**	**+Funding**	**?**	**-**
Kennedy 2002 [53]	**+**	**?**	**?**	**+**	**+**	**+Funding**	**?**	**-**
Kennedy 2007 [54]	**+**	**?**	**?**	**?**	**+**	**+Funding**	**?**	**-**
Moulton 2001 [55]	**-**	**?**	**+**	**+**	**+**	**+Funding**	**?**	**-**
Scholey 2002 [56]	**+**	**?**	**?**	**+**	**+**	**+Funding**	**?**	**-**
**Ginseng**								
Yeo 2012 [57]	**-**	**-**	**-**	**+**	**+**	**+Funding**	**-**	**?**
**Guarana**								
Haskell 2007 [58]	**+**	**?**	**?**	**+**	**+**	**+Funding**	**?**	**-**
Kennedy 2004 [59]	**+**	**?**	**?**	**+**	**+**	**+Funding**	**?**	**-**
Kennedy 2008 [60]	**-**	**+**	**+**	**+**	**+**	**+Funding**	**?**	**-**
Veasey 2015 [61]	**+**	**?**	**?**	**+**	**+**	**+Funding**	**?**	**-**
**Nitrate**								
Thompson 2014 [62]	**+**	**?**	**+**	**+**	**+**	**+Funding**	**+**	**+**
Wightman 2015 [63]	**+**	**+**	**+**	**+**	**+**	**+**	**+**	**++**
**Omega-3**								
Bauer 2014 [28]	**+**	**+**	**+**	**+**	**+**	**+Funding**	**+**	**++**
Giles 2015 [64]	**-**	**?**	**?**	**+**	**+**	**+Funding**	**?**	**-**
**Prebiotics**								
Smith 2015 [65]	**+**	**?**	**+**	**+**	**+**	**+Funding**	**+**	**+**
**Tyrosine**								
Colzato 2013 [66]	**+**	**?**	**?**	**+**	**+**	**+**	**?**	**-**
Colzato 2014 [67]	**+**	**?**	**?**	**+**	**+**	**+**	**?**	**-**
Colzato 2015 [68]	**+**	**?**	**?**	**+**	**+**	**+**	**?**	**-**
Coull 2015 [69]	**+**	**?**	**?**	**+**	**+**	**+**	**?**	**-**
Kishore 2013 [70]	**+**	**?**	**+**	**+**	**+**	**+**	**+**	**+**
Steenbergen 2015 [71]	**+**	**?**	**?**	**+**	**+**	**+**	**?**	**-**
Watson 2012 [72]	**-**	**?**	**+**	**+**	**+**	**+**	**?**	**-**
Jongkees 2017 [73]	**?**	**?**	**+**	**+**	**+**	**+**	**?**	**-**
**B Vitamins**								
Bryan 2002 [74]	**-**	**?**	**?**	**+**	**+**	**+**	**?**	**-**

**Risk of Bias Criteria:** -: Not done/poorly done; +: Done; ?: Unclear. **Risk of Bias Judgement:** -: High Risk; ++: Acceptable Risk; ?: Unclear Risk. **SIGN Quality Evaluation:** ++: High Quality; +: Acceptable Quality; -: Low Quality; ?: Very Low Quality.

**Table 3:** Summary of evidence of included studies.

Supplement/Author and Referenc	Population ^a^ (Sample Size (*n*), Age Range (mean ± SD), *n* = Male/Female)	Intervention (dose (Supplier), Placebo, Frequency (*f*))	Moderator Description	Outcome Summary Positive (+), Negative (-), Inconclusive (<>), Null (0)
*Beta-Alanine*	(*n* = 1 study)			
Hoffman et al. (2014) [39]	*n* = 20 M age = beta-alanine: 20.1 years (0.7); placebo: 20.2years(1.1) 20 Males; 0 Females	6 g beta-alanine tablet (CarnoSyn^TM^; Natural Alternatives International) Placebo (rice flour) *f* = 3/day (2 g/serve), 28 day	Fatigue (physical and cognitive)	Information processing speed (+) Memory (0)
*Caffeine*	(*n* = 7 studies)	subsequent from lit review		
Aidman et al. (2018) [40]	*n* = 11 M age = 22.5 years (2.7) 6 Males; 5 Females	800 mg of caffeine gum (Military Energy Gum) Placebo gum *f* = 200 mg 4/day, 2 hrly (0100–0700h), 2 days	Sleep deprivation (period)	Attention (+) Executive function (+)
Brunye et al. (2010) [41]	*n* = 36 M age = 20.1 years (ND) 10 Males; 26 Females	Caffeine capsule: 0, 100, 200 or 400 mg Placebo (capsule) *f* = single dose	Habitual caffeine intake	Executive function 400 mg only (+) Attention (+)
Hussain and Cole (2015) [42]	*n* = 26 M age = caffeine: 22.9 years (0.9); placebo: 24 years (0.8) 12 Males; 14 Females	200 mg caffeine placebo *f* = capsule, single dose	24-h recall	Memory (0) Executive function (0)
Kahathuduwa et al. (2017) [43]	*n* = 20 M age = 21.9 years (ND) 20 Males; 0 Females	160 mg caffeine drink placebo drink *f* = single dose 5-way crossover	No moderator	Memory (+) Executive function (+) Information processing speed (0)
Kamimori et al. (2015) [44]	*n* = 20 M age = 28.6 years (4.7) 20 Males; 0 Females	200 mg caffeine gum (Stay Alert^®^_)_ Placebo gum *f* = 2145, 0100, 0345 and 0700 h (total 800 mg/day), 3 days	3 nights of sustained wakefulness	Attention (+) Executive function (+)
Reyner and Horne (2000) [45]	*n* = 16 (2 studies); *n* = 8/study M age = 23 years (2) 8 Males; 8 Females	200 mg caffeine (2–3 cups of coffee) Placebo *f* = single dose	Restricted sleep (study 1) and sleep deprivation (study 2)	Attention (+) Sleep restriction only (Study 1)
Soar et al. (2016) [46]	*n* = 43 M age = 28.1 years 17 Males; 26 Females	1 cup (50 mg caffeine) of caffeinated coffee Placebo (decaffeinated) *f* = single dose	Habitual caffeine intake	Information processing speed (+) Executive function (<>) Memory (+)
*Flavonoids*	(*n* = 5 studies)			
Lamport et al. (2017) [47]	Study 1: *n* = 28 M age = 22 years (2.2) 4 Males; 24 Females Study 2: *n* = 16; M age = 22 years (1.9) 8 Males; 8 Females	70.5 mg (500 mL) flavonoid drink (Tropicana Ruby Breakfast Juice; PepsiCo Inc.;) placebo *f* = single dose	Time since ingestion	Information processing speed (+) Memory (0) Attention (0) Executive function (0)
Scholey et al. (2010) [48]	*n* = 30 M age = 21.9 years (0.6) 13 Males; 17 Females	Dairy cocoa drink (dose: 520 mg and 994 mg Cocoa Flavanols) placebo (nutrient-matched, low flavanol) *f* = single dose	High cognitive demand	Memory (<>) Information processing speed 994 mg only (+) at 30 and 40 min
Watson et al. (2015) [49]	*n* = 36 M age = 24.8 years (3.9) M: F not disclosed	525 ± 5 mg of polyphenols /60 kg body weight (anthocyanin-enriched blackcurrant extract; 1.66 g of DelCyan) or from 142 mL of blackcurrant fruit juice (Blackadder), Placebo (0 mg polyphenols) drink; *f* = single dose	No moderator	Attention (+)
Wightman et al. (2012) [50]	*n* = 27 M age = 22 years 11 Males; 16 Females	135 mg or 270 mg of epigallocatechin (green tea; DSM Nutritional Products) placebo (not disclosed) *f* = single dose (2 capsules)	No moderator	Information processing speed (0) Memory (0) Executive function (0)
*Gingko Biloba*	(*n* = 7 studies)			
Elsabagh et al. (2005) [51]	Study 1: *n* = 52 M age = gingko 21.3 years (0.3); Placebo 21.7years(0.4) 26 Males; 26 Females Study 2: *n* = 40 M age = gingko 21.2 years (0.3); Placebo 21.5 (0.3) 21 Males; 19 Females	120 mg of standardised gingko extract (LI 1370; Lichtwer Pharma) Placebo ND *f* = Study 1 single dose; Study 2 daily for 6 wk.	No moderator	Study 1: Attention (+) Memory (<>) Executive function (0) Study 2: Attention (0) Memory (0) Executive function (0)
Kennedy et al. (2002) [53]	*n* = 20; M age = 21.2 years (3.9) 5 Males; 15 Females	60 mg of gingko biloba (GK501, pharmaton), 100 mg P. ginseng extract (G115, Pharmaton), 160 mg ginkgo/ginseng combination (100 mg ginseng/60 mg ginkgo per capsule, Pharmaton) Placebo inert (ND) *f* = Single dose (6 capsules); 360 mg ginkgo, 400 mg ginseng, 960 mg ginkgo/ginseng, inert placebo	No moderator	Attention (<>) Memory (<>) Information processing speed (<>)
Kennedy et al. (2000) [52]	*n* = 20 M age = 19.9 years (ND) 2 Males; 18 Females	60 mg standardised gingko extract (GK501, Pharmanton,) Placebo inert (ND) *f* = Single dose (6 capsules); 120, 240, 360 mg gingko, or inert placebo	No moderator	Attention (<>) Memory (<>) Information processing speed (<>)
Kennedy et al. (2007) [54]	*n* = 28; M age = 20.4 years (1.2) 10 Males; 18 Females	120 mg standardised gingko biloba extract (60 mg ginkgo per capsule); complexed with 360 mg of phosphatidylserine OR 360 mg of phosphatidylcholine OR Placebo (Indena SpA, Milan) *f* = single dose (2 capsules)	Complexed extract with two phospholipids	Information processing speed (+); phosphatidylserine only Attention (0) Memory (<>)
Moulton et al. (2001) [55]	*n* = 60 M age = Gingko: 20.6y (1.9); placebo: 20.4 years (1.8) 60 Males; 0 Females	120 mg of BioGinkgo 27/7 (LI 1370) Placebo = fillers (Pharmanex Inc.) *f* = once daily (2 tablets), 5 days	No moderator	Memory (<>) Information processing speed (0)
Scholey and Kennedy (2002) [56]	*n* = 20 (study 1) M age = 19.9 years (1.5) 2 Males; 18 Females	120, 240, or 360 mg of standardized gingko biloba extract (GK501, Pharmaton SA, 60 mg ginkgo biloba/capsule) Placebo = ND *f* = single dose 6 capsules (60 mg/capsule)	Serial arithmetic tasks with different cognitive loads	Memory (<>)
*Ginseng*	(*n* = 1 study)	subsequent from lit review		
Yeo et al. (2012) [57]	*n* = 15 M age = ND15 Males; 0 Females	4500 mg/day of Korean red ginseng Placebo = ND *f* = 5 capsules (300 mg/capsule) 3 doses/day; 2 wk	No moderator	Attention (+); brain activity Memory (+); brain activity Information processing speed (0)
*Guarana / Guarana + Multivitamin*	(*n* = 4 studies)			
Haskell et al. (2007) [58]	*n* = 26 M age = 21.4 years (0.6) 8 Males; 18 Females	37.5, 75, 150 and 300 mg standardize guarana extract (PC-102, Pharmaton, SA) Placebo = ND *f* = single dose; 1 capsule/day; 6 days	No moderator	Memory (<>) Attention (0) Information processing speed (0)
Kennedy et al. (2004) [59]	*n* = 28 M age = 21.4 years (0.8) 9 Males; 19 Females	75 mg of a standardised guarana extract (Pharmaton) Placebo = ND *f* = single dose (2 capsules)	No moderator	Memory (<>) Attention (<>) Information processing speed (<>) Executive function (<>)
Kennedy et al. (2008) [60]	*n* = 130 M age = 20.9 years (1.6) 60 Males; 70 Females	Berocca Boost® multivitamin + mineral complex (222.2 mg guarana) Placebo = inert effervescent tablet *f* = single dose, effervesce tablet in 200 mL water	Cognitive demand	Attention (+) Memory (0) Information processing speed (<>) Executive function (<>)
Veasey et al. (2015) [61]	*n* = 40 M age = 21.4 years 40 Males; 0 Females	Berocca Boost® multivitamin + mineral complex (222.2 mg guarana) Placebo = inert effervescent tablet *f* = single dose, effervesce tablet in 250 mL water	Exercise	Attention (0) Memory (<>) Information processing speed (<>)
*Nitrate*	(*n* = 2 studies)			
Thompson et al. (2014) [62]	*n* = 16 M age = 24.4 years (4.0) 16 Males; 0 Females	5 mmol nitrate drink (450 mL beetroot juice, 50 mL low calorie blackcurrant cordial; James White Drinks, Ipswich UK) Placebo (50 mL blackcurrant cordial, 45 mL apple juice, 405 mL water) *f* = single dose	Mental fatigue and exercise intensities	Attention (0) Executive function (0)
Wightman et al. (2015) [63]	*n* = 40 Mean = 21.3 years (0.7) 13 Males; 27 Females	5.5 mmol nitrate drink (450 mL beetroot juice, 50 mL low calorie apple and blackcurrant cordial; James White Drinks, UK) Placebo (50 mL apple and blackcurrant cordial, 50 mL apple juice, 400 mL water) *f* = single dose	No moderator	Memory (<>) Attention (0)
*Omega-3*	(*n* = 2 studies)			
Bauer et al. (2014) [28]	*n* = 13 M age = 23.8 years (3.5) 4 Males; 9 Females	EPA-rich (590 mg EPA, 137 mg DHA; 4.3:1; Eye-Q_TM_, Novasel); DHA-rich (417 mg DHA 159 mg EPA; 3:1; Efalex^TM^, Efamol) Placebo—NONE *f* = 6/day, 30 days	No moderator	Executive function (+) Memory (<>)
Giles et al. (2015) [64]	*n* = 72 M age = Omega-3: 20.8 years (2.4); Placebo: 20.5 years (1.7) 27 Males; 45 Females	2800 mg fish oil (1680 mg EPA, 1120 mg DHA; Compound Solutions, CT Placebo (2800 mg olive oil; Compound Solutions, CT) *f* = 7 capsules/day, 35 days	Stress	Attention (0)
*Prebiotics*	(*n* = 1 study)			
Smith et al. (2015) [65]	*n* = 50 M age = 23.0 years (ND) 19 Males; 28 Females	5 g oligofructose-enriched inulin powder (ORAFTI, Tienen, Belgium) Placebo powder (ORAFTI, Tienen, Belgium) *f =* single dose, added to decaffeinated tea or coffee	No moderator	Memory (<>) Information processing speed (0) Attention (0) Executive function (0)
*Tyrosine*	(*n* = 8 studies)			
Colzato et al. (2013) [66]	*n* = 22 M age = 19.7 years (ND) 0 Males; 22 Females	2 g tyrosine (Bulk Powders Ltd.) Placebo 2 g microcrystalline cellulose (Sigma-Aldrich) *f* = single dose, dissolved in 400 mL of orange juice	Cognitive stress	Memory (<>)
Colzato et al. (2014) [67]	*n* = 22 M age = 20.4 years (ND) 0 Males; 22 Females	2 g tyrosine (Bulk Powders Ltd.) Placebo 2 g microcrystalline cellulose (Sigma-Aldrich LLR, Zwijndrecht, Netherlands *f* = single dose, dissolved in 400 mL of orange juice	No moderator	Executive function (<>)
Colzato et al. (2015) [68]	*n* = 32 M age = 19.4 years (ND) 8 Males; 24 Females	2 g tyrosine (Bulk Powders Ltd.) Placebo 2 g microcrystalline cellulose (Sigma-Aldrich) *f* = single dose, dissolved in 400 mL of orange juice	No moderator	Executive function (<>)
Coull et al. (2015) [69]	*n* = 8 M age = 21.0 years (1.0) 8 Males	Total of 150 mg/kg tyrosine (Myprotein.co.uk) mixed with 250 mL of sugar-free lemon squash (Tesco, UK) Placebo 250 mL of sugar-free lemon squash (Tesco, UK) *f* = single dose	Exercise in a hot environment	Vigilance (+)
Kishore et al. (2013) [70]	*n* = 10 M age = not stated 10 Males; 0 Females	100 mg/kg tyrosine 50 g low fat, high-energy bar (containing 6.5 g of L-tyrosine; Defence Food Research Laboratory, Defence Research and Development Organization, India) Placebo 50 g low fat, high-energy bar (Defence Food Research Laboratory, Defence Research and Development Organization, India) *f* = single dose	Heat stress	Brain Activity: Attention (+) Executive function (+)
Steenbergen et al. (2015) [71]	*n* = 22 M age = 19.3 years (1.5) 0 Males; 22 Females	2 g tyrosine (Bulk Powders Ltd.) Placebo 2 g microcrystalline cellulose (Sigma-Aldrich) *f* = single dose, dissolved in 400 mL of orange juice	No moderator	Executive function (+)
Watson et al. (2012) [72]	*n* = 8 M age = 23.0 years (3.0) 8 Males; 0 Females	Total 150 mg/kg tyrosine (SHS Intl., Liverpool, UK) in a sugar-free fruit drink (Tesco Ltd., Chestnut, UK) Placebo sugar-free fruit drink (Tesco Ltd., Chestnut, UK) *f* = 2 doses 30 min apart to yield total of 150mg/kg	Exercise in a warm environment	Attention (0) Memory (0) Executive function (0)
Jongkees et al. (2017) [73]	Study 1: *n* = 36 M age = Tyrosine 22.2 (2.4); Placebo 20.8 (1.9), 2 Males; 34 Females	2 g tyrosine (Bulk Powders Ltd.) Placebo 2 g microcrystalline cellulose (Sigma-Aldrich) *f* = single dose, dissolved in 400ml of orange juice	Study 1: No Moderator	Study 1: Memory (+)
*B Vitamins*	(*n* = 1 study)			
Bryan et al. (2002) [74]	*n* = 56 M age = 25.2 years (3.2) 0 Males; 56 Females	750 µg folate, 15 µg B_12_, 75 mg B_6_ capsule (Technical Consultancy Services, NSW, Australia) Placebo microcrystalline cellulose, calcium phosphate, soy polysaccharide and magnesium capsule (Technical Consultancy Services, Australia) *f* = single dose, 5 weeks	No moderator	Information processing speed (0) Memory (0) Executive function (0)

^a^ All studies used healthy participants who gave informed consent.

**Table 4:** Higher-order cognitive functions and associated lower-order cognitive functions used in this review.

Higher-Order Cognitive Function	Lower-Order Cognitive Function
Information Processing Speed	Simple Reaction Time (RT)
	Choice/Complex RT
Psychomotor	Fine motor control
	Hand-eye coordination
	Gross motor control
	(Marksmanship involves first two)
Attention/Vigilance	Selective Attention
	Sustained Attention (vigilance)
	Divided Attention
	Target Detection
Memory	Procedural
	Episodic
	Semantic
	Prospective
	Short-Term Memory
	Visual Discrimination
Executive Function	Running Memory
	Working Memory Map Reading (orienteering) Inhibitory Control:
	Self-Control (emotions)
	Resistance to Interference
	Response Inhibition
	Logical reasoning
	Planning
	Cognitive Flexibility: Verbal Reasoning
	Numerical Reasoning (math ability)
	Spatial Reasoning
	Problem Solving
	Task Switching
	Cognitive Shifting

**Table 5:** Summary of impacts of reviewed dietary supplements on cognitive domains (# of participants (# of studies)). Improvement (+), decrease (-), no change (<>), or uncertain (?).

	Cognitive Domain				
Dietary Supplement	Psychomotor	Information Processing Speed	Attention/ Vigilance	Memory	Executive Function
Beta-alanine	+(20(1))			<>(20(1))	
Tyrosine					
Stress					
Heat			+(10(1))		+(10(1)) ^a^
Cognitive				<>(22(1))	
Heat and Physical		<>(16(2))	?(16(2))	<>(8(1))	<>(8(1))
No Stress				+(36(1))	
Omega-3			?(85(2))	-(13(1))	?(85(2))
Vitamin B		-(56(1))		<>(56(1))	-(56(1))
Nitrate					
Stress					
Cognitive/Physical					<>(16(1))
No Stress			<>(40(1))		<>(40(1))
Caffeine					
Stress					
Sleep Deprivation			+(39(3))		+(31(2))
No Stress	+(43(1))	<>(20(1))	?(36(1))	?(46(2))	?(125(4))
Flavanoids		+(28(1))	+(36(1))	?(57(2))	<>(55(2))
Gingko biloba		?(120(4))	<>(48(2))	?(240(7))	?(52(1))
Ginseng		<>(15(1))	+(15(1))		
Guarana					
Stress					
Mental Fatigue		?(130(1))	+(130(1))	<>(130(1))	?(130(1))
Physical Fatigue		?(40(1))	<>(40(1))	?(40(1))	
No Stress		?(54(2))	?(54(2))	?(54(2))	?(28(1))
Prebiotics		<>(29(1))	<>(29(1))	?(29(1))	<>(29(1))

^a^ Effect seen for working memory and logical reasoning.

## Supplementary Materials

The following are available online at https://www.mdpi.com/2072-6643/12/2/545/s1, Table S1: Example of full search strategy used in the literature search; Table S2: Full table of evidence for included studies; Table S3: Excluded papers and reasons for exclusion; Table S4: PRISMA 2009 checklist; Table S5: GRADE analysis: Overall evidence synthesis for the impact of reviewed dietary supplements on cognitive domains.
