# Supplementary material for: A Systematic Review of the Effect of Dietary Supplements on Cognitive Performance in Healthy Young Adults and Military Personnel
Source: Nutrients. 2020 Feb 20;12(2):545. doi: 10.3390/nu12020545 (PMC7071459; doi:10.3390/nu12020545)
Supplement: Supplementary file 1 [file nutrients-12-00545-s001.pdf]

Table S1. Example of full search strategy used in the literature search.

Search 1

“meta analysis” OR “systematic literature review”
AND (“healthy young adults” OR army OR “armed forces” OR “special forces” OR military OR “elite military” OR operations OR deployed)
AND (“dietary supplements” OR “ergogenic aids” OR nutraceuticals OR nootropics OR pharmaceuticals)
AND (“performance enhancement” OR “cognitive enhancement” OR cognition OR attention OR memory OR “nutritional armour” OR “nutritional armor”)

Search 2

“meta analysis” OR “systematic literature review”
AND (“healthy young adults” OR army OR “armed forces” OR “special forces” OR military OR “elite military” OR operations OR deployed)
AND (“dietary supplements” OR “ergogenic aids” OR nutraceuticals OR nootropics OR pharmaceuticals)
OR (“performance enhancement” OR “cognitive enhancement” OR cognition OR attention OR memory OR “nutritional armour” OR “nutritional armor”)
AND (“adverse effects” OR “health outcomes” OR “health concerns” OR safety OR toxicity OR WADA)

Search 3

“meta analysis” OR “systematic literature review”
AND (“healthy young adults” OR army OR “armed forces” OR “special forces” OR military OR “elite military” OR operations OR deployed)
AND (“dietary supplements” OR “ergogenic aids” OR nutraceuticals OR nootropics OR pharmaceuticals)
OR (“performance enhancement” OR “cognitive enhancement” OR cognition OR attention OR memory OR “nutritional armour” OR “nutritional armor”)
AND (“adverse effects” OR “health outcomes” OR “health concerns” OR safety OR toxicity OR WADA)
OR Omega-3 OR Omega-6 OR probiotics OR prebiotics OR “gut-brain axis” OR caffeine OR WADA OR modafinil OR flavonoids OR vitamins OR “gingko biloba” OR bacopa OR curcumin OR ginseng OR protein OR tyrosine)

Search 4

“meta analysis” OR “systematic literature review”
AND (“healthy young adults” OR army OR “armed forces” OR “special forces” OR military OR “elite military” OR operations OR deployed)
AND micronutrients

Table S2. Full table of evidence for included studies

Reference	Design	Objectives	Population (sample size [n], age range [mean, SD], n = Male/Female)	Intervention (dose [supplier], placebo, frequency [*f*])	Moderator	Outcome measure(s)	Results
β-alanine	(n = 1 study)						
Hoffman et al. (2014)	Randomised double-blind, placebo-controlled, independent groups design	Examine the effect of beta-alanine supplementation in military personnel on physical and cognitive performance	n = 20; aged: ND (beta alanine, 20.1y, ± 0.7; placebo, 20.2y, ±1.1) Withdrawals: n = 2 (broken leg; no longer wished to participate); 20 Males; 0 Females	6 g beta-alanine tablet (CarnoSyn™; Natural Alterntaives International); Placebo (rice flour) *f* = 3/day (2 g dose), 28 days	Fatigue (physical & cognitive)	Military training (physical and complex cognitive tests) then modified Serial 7s	Cognitive assessments NS: (p = .844, partial η^2 = .003); Significant improvement in military specific cognitive tasks (marksmanship and speed) covering domains of information processing speed and memory ($p < .05$)
Caffeine	(n = 8 studies)						
Aidman et al. (2016, under review)	Randomised, double-blind, placebo-controlled, independent groups design	Examined if caffeine impacts the relationship between driving performance and drowsiness in sleep deprived drivers	n = 11; aged: 18-28y (22.5, ±2.7); "low caffeine users"; 6 Males; 5 Females	800 mg of caffeine gum (Military Energy Gum); Placebo gum; *f* = 4/d ay (200 mg dose), 2 hrly (0100–0700 h), 2 days	Sleep deprivation (period)	Stimulated driving performance: lane keeping (SDlat), speed maintenance (SDS); Drowsiness (JDS)	SDlat x condition x JDS ($p < .001$); SDS x condition x JDS (p = .027)
Brunye et al. (2010)	Randomised, double-blind, placebo-controlled,	Assessed how 4 doses of caffeine would affect habitual users'	n = 36; aged: ND (20.11y, ND); Withdrawals: n = 2 (did not finish ANT on 1+ session/s); "high caffeine	Caffeine capsule: 100, 200 or 400 mg Placebo capsule (0 mg caffeine); *f* = single dose	No moderator	ANT (alerting, orienting, executive control)	Alerting: "marginal effect" (p = .09, η^2 = .06); 400 mg better than placebo ($p < .05$, d = .36). Orienting: NS. Executive control: (p <

	crossover design; 3 day washout	performance on the ANT	consumers" (≥ 500 mg/d); 10 Males; 0 Females				.05, $\eta^2 = .10$); 400 mg better ($p < .01$, $d = .6$)
Gottselig et al. (2010) [#]	Randomised, double-blind, placebo-controlled, crossover design; 1 week washout	Examined whether sleep deprivation would impair RNG and if caffeine would mitigate this effect	n = 23; aged 20-30y (24.7, ND); Consumed < 300 mg caffeine/d Withdrawals: n = 2 (task non-compliance); 23 Males; 0 Females	200 mg caffeine or placebo *f* = 2 doses (11 and 23 hrs into sleep deprivation)	Sleep deprivation	RNG (executive function): no. of responses, rule violations, response redundancy, stereotypy of response pairs, counting tendency	No. of responses: session x treatment interaction: ameliorated effect of sleep dep on second day ($p < .001$);others: NS
Hussain & Cole (2015)	Double-blind, placebo-controlled, independent groups design; not randomised	Investigated if a single dose of caffeine immediately after visuomotor skill acquisition alters 24 h retention of the skill	n = 26; aged: ND (23.4 y, ND); "low caffeine consumers" (< 500 mg/wk); 12 Males; 14 Females	200 mg caffeine or placebo (capsule) *f* = single dose (administered after learning)	24 h recall	Learning rate, learning magnitude, retention magnitude (long term memory)	All: NS
Kahathuduwa et al. (2016)	Single-blind, placebo-controlled five-way crossover design; 1 day washout	Comparing acute effects of caffeine and theanine and their combination on attention	n = 20; aged 21-23y (21.9, ND); consumed < 4 cups of tea or coffee/d; 20 Males; 0 Females	160 mg caffeine and placebo (in distilled water; see text for other conditions); *f* = single dose	No moderator	SVRT, RVRT	SVRT: NS RVRT: improved ($p = .043$, $d = .486$)
Kamimori et al. (2015)	Randomised, double-blind, placebo-controlled, independent groups design	Test the effectiveness of repeated 200 mg doses of caffeine on cognition and live-fire marksmanship during 3 nights of	n = 20; aged: ND (28.6 y, ND); 20 Males (Special Forces); 0 Females; Varied caffeine intake	800 mg caffeine (Stay Alert® gum) or placebo *f* =4/day (200 mg/serve; 21:45 h,	Sleep deprivation	Vigilance (PVT, FVT), logical reasoning	PVT: (condition x time) $p < .02$ (caffeine mitigated declines); FVT: $p < .001$ (caffeine scored better); vigilance monitor: $p <$

		sustained wakefulness followed by 4 h afternoon sleep periods		01:00 h, 03:45 h, 07:00 h), 3 days (800 mg/d)			.001; logical reasoning: *p* < .001
Lieberman et al. (2002) [#]	Randomised, double-blind, placebo-controlled, independent groups design	Assessing if moderate doses of caffeine reduce adverse effects after 72 h sleep deprivation and stress on cognitive performance	n = 68; aged: ND (23.9 y, ND) Withdrawals: n = 22; 68 Male (US SEAL trainees); 0 Females;	100 mg, 200 mg, 300 mg of caffeine or placebo (capsule) *f* = single dose	Sleep deprivation	Attention (scanning visual vigilance, four-choice VRT), spatial working memory (matching-to-sample), motor learning, STM (repeated acquisition), mood; measured at 1 & 8 h post-dose	Vigilance: linear improvement with dose (targets detected $p = .046$;RT: $p = .042$); VRT: improved ($p = .049$); Match: NS; Acquisition: improved time to complete ($p = .019$); Fatigue: reduced at 1 h ($p = .02$); 8 h ($p = .04$)
Magill et al. (2003) [#]	Randomised, double-blind, placebo-controlled, independent groups design	Compare the effects of placebo versus D-amphetamine, caffeine, tyrosine and phentermine on cognitive and motor performance during long-term sleep deprivation	n = 76; aged 18-35 y (ND, ND); 76 Males; 0 Females	300 mg/70 kg of caffeine or placebo	Long-term sleep deprivation	Attention (visual scanning, visual vigilance, four-choice serial reaction time), memory (long-term memory), working memory (running memory task), executive function (logical reasoning, Stroop) mathematical processing, time wall task, pursuit tracking task, trails (B) task	Running memory, logical reasoning, mathematical processing, visual vigilance deteriorated with sleep dep., $p \leq .021$); Running memory RT, logical reasoning RT, maths processing RT, tracking error, vigilance hits, Stroop RT, trails B: $p \leq .0032$ at 1.5 h; Running memory RT, logical reasoning RT, maths processing RT, vigilance RT, vigilance hits, Stroop RT, 4-choice RT: $p \leq .01$ at 5.5 h

Reyner & Horne (2000)	Randomised, double-blind, placebo-controlled, crossover design; counterbalanced; 1 week washout	Examined the effect of caffeine on early morning driving ability after restricted sleep (Study 1) and no sleep (Study 2)	n = 16 (n = 8/study); aged: ND (23, 2); "moderate caffeine consumers (2-4 cups daily)"; 8 Males; 8 Females	200 mg caffeine or placebo (administered as decaf. coffee); *f* = single dose	Restricted sleep (study 1) and sleep deprivation (study 2)	Driving incidents, sleepiness	1. incidents: $p < .05$; sleepiness: $p < .0232$. incidents; incidents in first 30 mins: $p < .05$; sleepiness: *p* not provided
Soar et al. (2016)	Randomised, double-blind, placebo-controlled, crossover design; counterbalanced; 1 week washout	Assess if a caffeinated beverage (coffee) affects executive function as measured by the Stroop task and the more ecologically valid JEF	n = 43; aged: ND (28.1, ND); "regular caffeine users"; 17 Males; 26 Females	1 cup of caffeinated coffee (≈ 50 mg caffeine) or decaf. coffee (placebo); *f* = single dose	No moderator	Stroop task and JEF: planning, selection, prioritisation, creative thinking, adaptive thinking, action-, event-, & time-based prospective memory	Stroop (no. correct: NS; RT: caffeine faster, $p = .03$)<br>Planning: $p < .01$; creative thinking: $p < .001$; event-based PM: $p = .001$; time-based PM: $p = .04$; action-based PM: $p < .01$; overall JEF: $p < .001$;<br>others: NS
Flavonoids	(n = 5 studies)						
Lamport et al. (2017)	Randomised, single-blind, placebo-controlled, crossover design; 1 week washout	Examine whether flavanone-rich citrus juice is associated with acute cognitive benefits and increased regional CBF	Cognitive arm: n = 28; aged 18-30y (22, 2.2); 4 Males; 24 Females;<br>Withdrawals: n = 4 (work/illness)<br>ASL imaging arm: n = 16; aged 18-30y (22 y, 1.9); 8 Males; 8 Females;<br>Ps did not take part in both arms	500 mL high flavanone drink (Tropicana Ruby Breakfast Juice; PepsiCo Inc.; 70.5 mg flavonoids) and placebo (0 mg flavonoids); *f* = single dose	No moderator	Immediate and delayed word recall, logical memory (immediate and delayed recall), sequence learning task, DSST, Stroop, letter memory test, Go/No Go, spatial delayed recall	DSST: drink x time interaction ($p < .01$); others: NS

Scholey et al. (2010)	Randomised, double-blind, placebo-controlled, three-period crossover design; counterbalanced; 3 day washout	Examine if cocoa flavanols (CF) can modulate declines in cognitive performance resulting from high cognitive demand	n = 30; aged 18-35 y (21.9, 0.6); 13 Males; 17 Females	Dairy-based cocoa drinks containing 520 mg and 994 mg of CF, and a nutrient-matched, low flavanol control drink; *f* = single dose	High cognitive demand	Serial subtraction tasks (Serial 3s and 7s), RVIP, mental fatigue	Serial 3s ($p < .001$, 520 mg best; errors: $p = .01$); Serial 7s (NS; errors: 994 mg had more errors at 30 & 40 mins, $p \leq .02$); RVIP (accuracy: NS; RT: 994 mg faster at 30 & 40 mins, $p \leq .02$; false alarms: NS); fatigue: 520 mg less fatigued ($p \leq .006$) at 10, 20, 40, 50, & 60 mins
Watson et al. (2015)	Randomised, double-blind, placebo-controlled, crossover design; counterbalanced; 1 week washout	Explore the effects of two blackcurrant extracts (with balanced polyphenol content) on attention, mood, and biochemical parameters (peripheral monoamines, prolactin, and blood glucose)	n = 36; aged 18-34y (24.8, 3.9); Male:Female: ND	525 ± 5 mg of polyphenols /60 kg bodyweight from an anthocyanin-enriched blackcurrant extract (1.66 g of DelCyan) or from 142 mL of blackcurrant fruit juice (Blackadder), or placebo (0 mg of polyphenols) administered as a drink; *f* = single dose	No moderator	Digit vigilance, Stroop, RVIP (administered 7 times in the fashion of a cognitive demand battery), logical reasoning (executive function), mood, mental fatigue	Digit vigilance (accuracy: NS, condition x repetition RT: $p = .044$); Stroop: NS; RVIP (accuracy: $p = .005$); mood (alertness: $p =.02$ for DelCyan compared with control; juice NS); fatigue: improved at 70 mins for DelCyan ($p = .046$)
Wightman et al. (2012)	Randomised, double-blind, placebo-controlled, crossover design; counterbalanced; 1 week washout	Assess the effects of EGCG on CBF in the frontal cortex, and on cognitive performance	n = 27; aged 18-30 y (22, ND); Substitutions: n = 5 (failed to complete testing and replaced during data collection); 11 Males; 16	135 mg or 270 mg of EGCG (DSM Nutritional Products) or placebo (ND); *f* = single dose (2 capsules)	No moderator	Simple reaction time, oddball reaction time, serial 3s, serial 7s, RVIP, Stroop, executive function/working memory, attention	All: NS

			Females				
Ginko biloba	(n = 7 studies)						
Elsabagh et al. (2005)	Randomised, double-blind, placebo-controlled independent groups design	Compare the effects of gingko after acute and chronic treatment on tests of attention, memory, and executive function	Study 1: n = 52; aged 18-26y (gingko: 21.3, 0.3; placebo: 21.7, 0.4); 26 Males; 26 Females Study 2: n = 40; aged 18-26y (gingko: 21.2 , 0.3; placebo: 21.5, 0.3); 21 Males; 19 Females	120 mg of standardised gingko extract (LI 1370; 25% total gingko flavonoids; Lichtwer Pharma one-a-day tablets) or matched placebo *f* = 1. single dose (1 tablet); 2. 1/d, 6 wks	No moderator	Sustained attention (serial addition), episodic memory (PRM & SRM, delayed word and picture recall), spatial WM, executive function (mental flexibility and planning)	1. attention (condition x speed: $p < .02$; differed at baseline); episodic memory (PRM: $p = .05$; SRM: NS; word & picture recall: NS); spatial WM: NS; executive function: NS 2. All: NS
Kennedy et al. (2000)	Randomised, double-blind, placebo-controlled, crossover design; counterbalanced; 1 week washout	Examine whether acute gingko biloba administration influences speed/ accuracy of attention, and speed/quality of memory	n = 20;aged 19-24 y(19.9, ND); 2 Males; 18 Females	120 mg, 240 mg, & 360 mg of a standardised gingko extract (GK501; 24% gingko flavone glycosides) or matched placebo; *f* = single dose (6 capsules)	No moderator	QoM, SoM, SoA, QoA; measured at baseline, 1, 2.5, 4 & 6 h post-dose)	QoM: 120mg ($p \leq .033$ at 1 & 4 h); SoM: 360mg ($p = .04$ at 2.5 h) & 240 mg deteriorated ($p = .03$ at 4h); SoA (at 2.5, 4 & 6 h): 240 mg ($p \leq .036$) & 360mg ($p < .001$); QoA: 240 mg ($p = .045$ at 1 h)
Kennedy et al. (2002)	Randomised, double-blind, placebo-controlled, crossover design; counterbalanced; 1 week washout	Investigate the comparative cognitive effects of single doses of gingko, ginseng, and gingko/ginseng combination	n = 20; aged ND(21.2 y, 3.9); 5 Males; 15 Females	360 mg of gingko biloba extract (GK501) or matched placebo; *f* = single dose (6 capsules)	No moderator	QoM, SM, WM, SoM, SoA, QoA, serial 3s, serial 7s	QoM: $p = .008$ at 6 h; SM: $p \leq .032$ at 1 h & 6 h; serial 3s (responses: NS; errors: $p = .029$): serial 7s (responses: $p \leq .023$ at 4 h & 6 h); WM: NS; SoM: NS; SoA: NS; QoA: NS;significant

							improvement on individual tests in QoA
Kennedy et al. (2007)	Randomised, double-blind, placebo-controlled, crossover design; counterbalanced; 1 week washout	Assess the comparative cognitive effects of a low dose of gingko and products complexing the same extract with 2 phospholipids	n = 28; aged ND (20.4 y, 1.2); 10 Males; 18 Females	120 mg standardised gingko biloba extract, (120 mg of gingko complexed with 360 mg of phosphatidylserine, 120 mg of gingko complexed with 360 mg of phosphatidylcholine) or placebo (Indena SpA, Milan); *f* = single dose (2 hard-gelatine capsules)	No moderator	QoM, SM, WM, SoM, SoA, QoA, serial 3s, serial 7s	QoM (gingko with phosphatidylserine): $p \leq .02$ at 2.5 & 4 h, (gingko with phosphatidylcholine): $p = .008$ at 2.5 h, (gingko): NS; SoM (gingko with phosphatidylserine): $p \leq .011$ (all time points), (gingko): NS; other measures: NS
Moulton et al. (2001)	Double-blind, placebo-controlled, independent groups design; no baseline	Investigate possible effects of gingko biloba on memory	n = 60; aged ND (20.6, 1.9) 60 Males; 0 Females	120 mg of BioGinkgo (LI 1370) or placebo; *f* = single dose (2 tablets), 5 ds	No moderator	RT, memory (Sternberg memory scanning test, prose recall, reading span), WM (digit span)	Sternberg memory scanning (group x size x decision): $p < .05$; others: (RT, reading span, digit span, prose recall): NS
Scholey & Kennedy (2002; study 1)	Randomised, double-blind, placebo-controlled, crossover design; 1 week washout; counterbalanced	Examines acute effects of gingko, ginseng, and their combination on serial arithmetic tasks with different cognitive loads	n = 20; aged 19-24y (19.9, 1.5); 2 Males; 18 Females; same Ps as in Kennedy et al., 2000	120 mg, 240 mg, or 360 mg of gingko biloba extract (GK501, Pharmaton SA) or placebo; *f* = single dose (6 capsules)	No moderator	Serial 3s, serial 7s	Serial 3s (120 mg): $p < .05$ at 4 h, (240 mg): $p < .001$ at 4 h, (360 mg): $p < .05$ at 4 & 6 h; serial 3s errors (120 mg): increased, $p < .01$ at 4 h; serial 7s: NS; serial 7s errors (all doses): $p < .05$ at 2.5 h
Ginseng	(n = 1 study)						

Kennedy et al. (2001) #	Randomised, double-blind, placebo-controlled, crossover design; counterbalanced; 1 week washout	Investigate the effect of acute administration of ginseng on four aspects of cognitive performance	n = 20;aged 20-27 y(21.3, ND); 6 Males; 14 Females	200, 400 & 600 mg of ginseng extract (G115, Pharmaton SA) or matched placebo; *f* = single dose (6 capsules)	No moderator	QoM, SM, WM, SoM, SoA, QoA	SoA (200 mg & 600 mg): reduced, $p < .002$ (at 4 & 6 h), (400 mg): NS; QoA: (200 mg): $p = .048$ (at 6 h), (400 mg & 600 mg): NS; QoM (400 mg): $p \leq .035$ (at all time points), (600 mg): $p = .002$ (at 6 h), (200 mg): NS; SoM (400 mg): decreased, $p = .005$, (200 mg & 600 mg): NS; SM (400 mg): $p \leq .013$ (at all time points), (600 mg): $p \leq .046$ (at 1, 2.5 & 4 h), (200 mg): $p = .039$ (at 4 h); WM: NS
Kennedy et al. (2002) #	Randomised, double-blind, placebo-controlled, crossover design; counterbalance; 1 week washout	Investigate the comparative cognitive effects of single doses of gingko, ginseng, and gingko/ginseng combination	n = 20; aged ND(21.2 y, 3.9); 5 Males; 15 Females	400 mg of P. ginseng extract (G115) or matched placebo; *f* = single dose (6 capsules)	No moderator	QoM, SM, WM, SoM, SoA, QoA, serial 3s, serial 7s	QoM: $p = .015$ at 4 h; SM: $p \leq .019$ at 4 h & 6 h; WM: NS; SoM: $p = .029$ at 4 h; SoA: NS; QoA: $p = .004$ at 2.5 h; serial 3s: NS; serial 7s: NS
Kennedy et al. (2004) #	Randomised, double-blind, placebo-controlled, crossover design; counterbalance; 1 week washout	Assess the cognitive effects of separate doses of guarana, ginseng, and their combination	n = 28; aged 18-24 y(21.4, 0.8); 9 Males; 19 Females	200 mg of standardised ginseng extract (G115) or placebo; *f* = single dose (2 capsules)	No moderator	SoA, AoA, SoM, SM,WM, logical reasoning, sentence verification, serial 3s, serial 7s	SoA: $p \leq .04$ at 4 & 6 h; AoA: NS; SoM: $p \leq .03$ at 1 & 4 h; SM: $p = .04$ at 2.5 h; WM: NS; logical reasoning: NS; sentence verification: $p \leq .007$ at 1, 2.5, 4 & 6 h; serial 3s: NS; serial 7s: $p \leq .024$ at 1 & 6 h

Reay et al. (2005) #	Randomised, double-blind, placebo-controlled, crossover design; counterbalance; 1 week washout	Examine the effect of single doses of ginseng on blood glucose levels and cognitive performance during sustained 'mentally demanding' tasks (CDB)	n = 30; aged ND (22.6, ND), 14 Males; 16 Females	200 mg or 400 mg of P. ginseng (G115) or placebo; *f* = single dose (4 capsules)	Cognitive Fatigue	Serial 3s, serial 7s, RVIP, blood glucose	Serial 7s (200 mg): $p \leq .041$ (at 1, 2, 3, 4 & 6 CDB trials), (400 mg): NS; serial 3s: NS; RVIP: NS
Reay et al. (2006) #	Randomised, double-blind, placebo-controlled, crossover design; counterbalanced; 1 week washout	Investigate the relationship between the administration of both glucose and ginseng on cognition and blood glucose levels during sustained 'mentally demanding' tasks (CDB)	n = 27; aged ND(21.9, ND;17 Males; 10 Females	200 mg ginseng (G115), 25 mg glucose, 200 mg ginseng/25 mg glucose combination or placebo (25 mg glucose dissolved in 20 ml sugar-free cordial in 180 ml water); *f* = single dose (2 capsules)	Cognitive Fatigue	Serial 3s, serial 7s, RVIP, blood glucose	Serial 3s (ginseng): $p \leq .039$, (glucose): $p \leq .032$ (at 3, 4 & 6 CDB trials), (combination): NS; RVIP false alarms (glucose): $p \leq .02$ (at 2, 3 & 6 trials), (combination): $p \leq .006$ (at 3 & 6 trials), (ginseng): $p = .005$ (at 6 trial); serial 7s: NSat 1 h, $p = .046$, (glucose & ginseng/glucose combination): increased, $p < .001$
Reay et al. (2010) #	Randomised, double-blind, placebo-controlled, crossover design; counterbalanced; 6 day washout	Assess effects of ginseng on mood and working memory following a single dose and following sub-chronic (7 day) ingestion	n = 30; aged ND (22.9, ND Withdrawals: n = 28: Corsi block task & delayed word recognition; n = 24: N-back task; n = 23: random number generation task	200 mg or 400 mg of P. ginseng (G115) or matched placebo *f* = single dose (4 capsules), 7 ds	No moderator	Working memory (Corsi block task, N-back), executive function/inhibition (random number generation task)	3-Back RT (400 mg): $p = .023$, $d = .3$ (day 1, 2.5 h), and $p = .001$, $d = .5$ (day 8, 2.5 h), (200 mg): slower, $p <.05$, $d = .3$-$.6$ (day 1, 1, 2.5 & 4 h); 3-Back accuracy (400 mg): $p <.001$, $d = .7$-$.9$ (day 1, 1, 2.5 & 4 h), $p <.001$, $d = .6$-$.9$ (day 8, 1, 2.5 & 4 h), (200 mg):

							impaired, *p* = .02, *d* = .5 (day 8, 4 h); other: NS
Scholey & Kennedy (2002) [#]	Randomised, double-blind, placebo-controlled, crossover design; 1 week washout; counterbalanced	Examines acute effects of gingko, ginseng, and their combination on serial arithmetic tasks with different cognitive loads	n = 20; aged 20-27y (21.3, ND; 6 Males;; 14 Females same Ps as in Kennedy et al., 2001	200 mg, 400 mg, or 600 mg of ginseng extract (G115, Pharmaton SA) or placebo; *f* = single dose (6 capsules)	Cognitive Fatigue	Serial 3s, serial 7s	Serial 3s: NS; serial 7s (200 mg): fewer subtractions at 1, 2.5 & 6 h (*p* <.05), (400 mg): improved accuracy at 4 & 6 h (*p* <.05), (200 mg): improved accuracy at 4 h (*p* <.05)
Yeo et al. (2012)	Randomised, double-blind, placebo-controlled, independent groups design	Investigate the effects of Korean red ginseng on cognitive performance in healthy young adults	n = 15;aged 19-25 y (ND, ND; 15 Males; 0 females nonparametric statistics were used due to small sample size	4500 mg of Korean red ginseng or placebo () *f* = 3 doses/d (5 capsules, 1500 mg/dose), 2 wks	No moderator	Vigilance, reaction time; P300 ERP	All cognitive measures: NS; ERP: C3 latency decreased (*p* = .033)
Guarana / guarnana+multivitamin	(n = 4 studies)						
Haskell et al.	Randomised, double-blind, placebo-controlled study; counterbalanced	Investigate acute, dose-related effects of guarana extract on cognition and mood in healthy young adults	n =26; aged ND (21.4, 0.6); 8 Males; 18 Females	37.5, 75, 150 and 300 mg guarana (Guarana extract PC-102, Pharmaton, SA) or matched placebo; *f* = single dose (1 capsule)per test day + baseline test (total 6 tests)	No moderator	SoA, AoA, SoM, SM, WM	SM enhanced (75mg dose p=.003, 35 mg dose p=.03)

Kennedy et al. (2004)	Randomised, double-blind, placebo-controlled, crossover design; counterbalanced; 1 week washout	Assess the cognitive effects of separate doses of guarana, ginseng, and their combination	n = 28; aged 18-24 y (21.4,0.8); 9 Males; 19 Females	75 mg of a standardised guarana extract or placebo; *f* = single dose (2 capsules)	No moderator	SoA, AoA, SoM, SM, WM, logical reasoning, sentence verification, serial 3s, serial 7s	SoA: $p \leq .025$ at 1, 4 & 6 h; AoA: NS; SoM NS; SM: $p = .002$ at 2.5 h; WM: NS; logical reasoning: NS; sentence verification: $p \leq .03$ at 2.5 & 4 h; serial 3s: NS, (errors): $p < .05$ at 2.5 & 4 h; serial 7s: $p \leq .05$ at 1, 2.5, 4 & 6 h, (increased errors): $p = .032$ at 4 h
Kennedy et al. (2008)	Randomised, double-blind, placebo-controlled, independent groups design	Assess acute effects of a vitamin/ mineral/guarana supplement on cognition during an extended period of cognitively demanding task performance	n =130; aged 18-24 y (20.9, 1.6); 60 Males; 70 Females	Berocca Boost® multivitamin and mineral complex with 222.2 mg guarana or placebo; *f* = single dose (effervesce tablet in 200 mL water)	Increased cognitive demand	Cognitive demand battery (serial 3s & 7s RVIP	RVIP (RT): $p = .025$, (accuracy) $p < .001$; serial 3s & 7s: NS
Veasey et al. (2015)	Randomised, double-blind, placebo-controlled, crossover design; counterbalance; 1 week washout	Examine the effect of a vitamin/ mineral/guarana supplement consumed prior to exercise on cognitive performance	n = 40; aged ND; (21.4, ND); Withdrawals: n = 3 pre randomisation); 40 Males; 0 Females	Berocca Boost® multivitamin and mineral complex with 222.2 mg guarana or placebo; *f* = single dose (effervesce tablet in 250 mL water)	Exercise	Attention (choice RT; RVIP), episodic memory (word recall, word and picture recognition), WM (numeric)	Choice RT & RVIP: NS; word recall & recognition: NS; picture recognition (accuracy) NS, (RT) $p = .0496$, $d = 0.40$; numeric WM: $p < .001$, $d = 0.71$
Nitrate	(n = 2 studies)						

Thompson et al. (2014)	Randomised, double-blind, placebo-controlled, crossover design; 1 week washout; counterbalanced	Investigate the effect of dietary nitrate on cerebral and skeletal muscle oxygenation, physiological measures, mental fatigue and cognitive performance at rest and over a range of exercise intensities	n = 16; aged (24.4, 4.0); 16 Males; 0 Females	500 mL of a nitrate supplement (450 mL beetroot juice containing 5 mmol nitrate, 50 mL low calorie blackcurrant cordial) or placebo (50 mL blackcurrant cordial, 45 mL apple juice, 405 mL water); *f* = single dose	Mental fatigue and exercise intensities	Attention (RVIP), executive function (Stroop)	Both cognitive measures: NS
Wightman et al. (2015)	Randomised, double-blind, placebo-controlled, independent groups design	Assess the effects of dietary nitrate on cognition and the CBF haemodynamic response in the prefrontal cortex during cognitive tasks	n = 40;aged 18-27 y (21.3, 0.7); 13 Males; 27 Females	500 mL of a nitrate supplement (450 mLbeetroot juice containing 5.5 mmol nitrate, 50 mL low calorie apple and blackcurrant cordial) or placebo (50 mLapple and blackcurrant cordial, 50 mL apple juice, 400 mL water); *f* =single dose (drunk in 3 portions over consecutive 10 minute periods)	No moderator	Cognitive battery (serial 3s serial 7s, RVIP) repeated 6 times	Serial 3s: $p < .01$ (however nitrate underperformed placebo at baseline)<br>Serial 7s, RVIP: NS
Omega-3	(n = 2 studies)						
Antypa et al. (2009) #	Randomised, double-blind, placebo-	Examine the effects of omega-3 on depression-related	n =56; aged (22.4, ND); Withdrawals: n = 2 (difficulty	2.3 g of omega-3 (1.74 g EPA, 0.25 g DHA; MorEPA™) or	No moderator	Attention (Go/NoGo); memory (immediate and delayed recall);	Attention, memory and emotional-info-processing tasks: NS;

	controlled, independent groups design	cognition in healthy adults	swallowing capsules, personal issues), 5 outliers excluded ; 10 Males; 46 Females	placebo; *f* = 3 doses/day (capsules); 4weeks		decision making (gambling task); emotional-info-processing tasks (affective Go/NoGo; facial expression recognition)	gambling task: NS effects of treatment (except in 'gains only' trials: omega-3 group were more risk-seeking, p =.03)
Bauer et al. (2011) #	Randomised, double-blind, crossover design; counterbalance; 30-day washout	Evaluate whether 2 omega-3 fatty acids (differing in EPA and DHA levels) alter cortical brain functioning, and performance on visual mental processing tasks	n = 34;aged 19-34 y (24.6, ND); Withdrawals: n = 12 (supplementation non-compliance; incomplete testing); 7 Males; 15 Females	EPA-rich formulation (590 mg EPA, 137 mg DHA; 4.3:1; Eye-Q TM, Novasel) and DHA-rich formulation (417 mg DHA 159 mg EPA; 3:1; Efalex TM, Efamol); *f* = 2 doses (3 capsules) / day (3 morning, 3 night); 30-days	No moderator	Mental processing (simple and choice motor reaction time, SMRT and CMRT respectively), Multifocal Visual Evoked Potentials (mfVEP)	SMRT: NS; CMRT: p = .03 (reduction in RT for EPA-rich supplement from baseline); EPA rich: and P1, p=.045. DHA rich: reduced amplitude N1, p=.034; P1 ns
Bauer et al. (2014)	Randomised, double-blind, crossover design; counterbalanced; 30-day washout; baseline measure (no placebo)	Investigate the effects of EPA-rich and DHA-rich supplementation on cognitive performance and functional brain activation	n = 13;aged 20-34 y (23.8, 3.5); Withdrawals: n = 2; 2 outliers excluded from analysis; 4 Males; 9 Females; Subset of the Ps from Bauer et al., 2011	EPA-rich formulation (590 mg EPA, 137 mg DHA; 4.3:1; Eye-Q TM, Novasel) and DHA-rich formulation (417 mg DHA 159 mg EPA; 3:1; Efalex TM, Efamol); *f* = 2 doses (3 capsules) / day (3 morning, 3 night); 30-days	No moderator	Stroop, spatial working memory	Stroop RT (congruent): p = .04 (EPA decreased RT compared to DHA); spatial working memory: NS
Benton et al. (2013) #	Randomised, double-blind, placebo-controlled,	Monitoring cognition and mood in healthy young adults	n = 305; aged (21.8, ND); Withdrawals: n = 20; 0 Males; 305	400 mg DHA (no EPA or other long-chain PUFA) or placebo; *f* = single dose (4	No moderator	Immediate and delayed word recall, recall of capitals, simple and choice reaction times,	Word recall (condition x time x immediate/delayed recall): p < .01 (limitations

	independent groups design; counterbalanced	supplemented with DHA	Females	capsules); 50 days (cognitive performance assessed at baseline, 25 and 50 days)		RVIP, visual acuity, mood	with this result); other measures: NS
Giles et al. (2015)	Randomised, double-blind, placebo-controlled, independent groups design	Evaluate the influence of omega-3 on stress-induced changes to mood, cognition and physiological stress markers	n = 72; aged (20.7, 2.4); 27 Males; 45 Females	2800 mg fish oil (1680 mg EPA + 1120 mg DHA) or placebo; f =single dose(7 capsules);35 days	Stress induced	Attentional control/emotional regulation (Emotional Interference Task; EIT)	EIT: NS
Jackson et al. (2012a) [#]	Randomised, double-blind, placebo-controlled, independent groups design	Evaluate the effects of 12 weeks of supplementation with DHA- and EPA-rich fish oil on cognitive function in healthy young adults	n = 159; aged 18-35 y (22, ND); Withdrawals: n = 19 (9 did not attend baseline session, 8 excluded for high oily fish intake, 2 illness, moving home); 46 Males; 113 Females	1 g DHA-rich (450 mg DHA + 90 mg EPA; 5:1) or EPA-rich (300 mg EPA + 200 mg DHA; 3:2) fish oil (EPAX AS) or placebo; f = single dose (2 capsules);12 weeks	No moderator	Simple RT; attention (choice and four-choice RT); episodic memory (immediate and delayed word recall, delayed word and picture recognition, names-to-faces recall); working memory (numeric, alphabetic, and telephone number, Corsi blocks, three-back); executive function (Stroop, verbal fluency); 3 x CDB (serial 3s and 7s, RVIP, mental fatigue)	Stroop (RT): $p = .014$, $r = 0.26$ (DHA faster than placebo); names-to-faces recall: DHA- and EPA-rich matched fewer items ($p = .047$, $r = 0.21$ and $p = .013$, $r = 0.26$, respectively); other: NS; Mental fatigue: $p = .009$, $r = 0.27$ (EPA-rich lower than placebo)
Jackson et al. (2012b) [#]	Randomised, double-blind, placebo-controlled,	Investigate the effects of 12 weeks of supplementation with DHA-rich fish	n = 65; aged 18-29 y (20.58 , ND); Withdrawals: n = 1 (illness), n = 2 NIRS	1 g (450 mg DHA + 90 mg EPA; 5:1) or 2 g (900 mg DHA + 180 mg EPA; 5:1) of	No moderator	Simple RT; attention (choice and four-choice RT; RVIP); working memory (numeric,	Choice RT: $p = .045$ (faster than placebo: 1g $p < .05$, $d = 0.72$; 2g $p < .05$, $d = 0.69$);

	independent groups design;no baseline measure	oil or placebo on CBF in healthy adults	data lost (fire drill); 16 Males; 49 Females	DHA-rich fish oil (EPAX AS) or placebo; *f* = single dose (4 capsules); 12 weeks		Corsi blocks, three-back, serial 7s); executive function (Stroop)	RVIP (RT): 2g *p* = .002, *d* = 0.93;other: NS
Karr et al. (2012) [#]	Randomised, double-blind, placebo-controlled, independent groups design	Assess the effect of omega-3 fish oil capsules on affect, executive control, inhibition, and verbal learning and memory in a college-age sample	n = 43; aged (20.5, ND); Withdrawals: n = 2 (supplementation non-compliance); 11 Males; 32 Females	480 mg DHA + 720 mg EPA or placebo; *f* = single dose (2 capsules);4 weeks	No moderator	Verbal learning and memory (RAVLT), inhibition (SCWT), executive control (TMT)	RAVLT stages 1-5: NS; stages 6 & 7: NS, condition x time (p .04, η^2 = .10; p .02, η^2 = .13 respectively) note, placebo declined; SCWT: NS; TMT: time (p .03, η^2 = .13) condition (p .02, η^2 = .14; placebo improved more than fish oil(practice effects)
Prebiotics	(n = 1 study)						
Smith et al. (2015)	Double-blind, placebo-controlled crossover design; 1-week washout; counterbalanced	Examine acute effects of oligofructose-enriched inulin added to a breakfast (cereal and toast with tea or coffee) on cognitive performance	n = 47;aged 19-30 y (23, ND); Withdrawals: n = 3; 19 Males; 28 Females	5 g oligofructose-enriched inulin (ORAFTI, Tienen, Belgium) or placebo; *f* =single dose (powder added to decaffeinated tea or coffee)	No moderator	Simple reaction time, attention (choice reaction time, categoric search task, repeated-digits vigilance task), memory (immediate/delayed free recall, delayed recognition, semantic processing, spatial memory), executive function (logical reasoning)	Free recall (immediate): p <.001, (delayed): p <.05; delayed recognition (accuracy): p <.05, (RT slower): p = .05; Logical reasoning, semantic processing, spatial memory, simple RT, choice RT, categoric search task, repeated-digits: NS

Tyrosine	(n = 8 studies)						
Colzato et al. (2013)	Randomised, double-blind, placebo-controlled, crossover design; counterbalanced; 3 to 7-day washout	Investigate whether the "updating" of working memory representations can be promoted by acute tyrosine supplementation	n = 22; aged 18-25 y (19.7, ND); 0 Males; 22 Females	2 g tyrosine (Bulk Powders Ltd.) or Placebo 2 g microcrystalline cellulose (Sigma-Aldrich); *f* = single dose (dissolved in 400ml of orange juice)	No moderator	Working memory (N-back task: "undemanding" – 1-back, "demanding" – 2-back)	Load x group (false alarms): $p = .02$, $\eta^2_p = 0.23$, (correct rejections): $p = .01$, η^2_p = NA, (accuracy): $p = .003$, $\eta^2_p = 0.35$<br>- 2 back task (tyrosine): (false alarms): $p = .03$, (correct rejections) $p = .02$, (accuracy) $p = .02$
Colzato et al. (2014)	Randomised, double-blind, placebo-controlled, crossover design; counterbalanced; 3 to 7-day washout	Assess the acute effect of tyrosine supplementation on the inhibition of behavioural responses	n = 22; aged (20.4, ND); 0 Males; 22 Females	2 g tyrosine (Bulk Powders Ltd.) or placebo 2 g microcrystalline cellulose (Sigma-Aldrich); *f* = single dose (dissolved in 400ml of orange juice)	No moderator	Inhibitory control (stop-signal task)	Tyrosine improved Stop-signal RT: $p < .05$, $\eta^2_p = 0.217$; go-signal RT: NS
Colzato et al. (2015)	Randomised, double-blind, placebo-controlled, crossover design; counterbalanced; 7-day washout, ± 1 day	Investigate the link between tyrosine supplementation and two creativity tasks tapping into convergent and divergent thinking	n = 32; aged (19.4, ND) ; 8 Males; 24 Females	2 g tyrosine (Bulk Powders Ltd.) or placebo 2 g microcrystalline cellulose (Sigma-Aldrich); *f* = single dose (dissolved in 400ml of orange juice)	No moderator	Convergent thinking (RAT), divergent thinking (AUT)	RAT: $p = .002$, $\eta^2_p = 0.30$, $d = 0.5$; AUT: NS<br>- tyrosine group performed better than placebo in RAT compared to baseline
Coull et al. (2015) [#]	Randomised, double-blind, placebo-controlled,	Examine the effect of tyrosine on cognitive and physical	n = 8; aged (21, ND); 8 Males; 0 Females	150 mg/kg / body wt of tyrosine (Myprotein.co.uk) mixed with placebo	Exercise in a warm environment	Attention (vigilance, dual-task test)	Vigilance (hits): $p = .015$, (misses): $p = .013$; dual-task test: NS

	crossover design;1-week washout; no baseline	performance during exercise in a warm environment		drink ; placebo 250 mL of sugar-free lemon squash; *f* = 300 mg/kg dose;5h and 1h pre-exercise			
Coull et al. (2016)	Study 1:Randomised, double-blind, placebo-controlled, independent groups design<br>Study 2: Double-blind, placebo-controlled, crossover design; both studies counterbalanced	Study 1: Assess the pharmacokinetics of 2 doses of tyrosine over an 8 h period<br>Study 2: Test the effects of the "optimal" dose of tyrosine on cognition, exercise and time trial performance in a hot environment	Study 1: n = 21; aged 19-26 y (M, ND); 21 Males; 0 females<br>Study 2: n = 8; aged (23, ND); 4 recruited from study 1; 8 Males; 0 Females	Study 1: 150 mg/kg or 300 mg/kg tyrosine (Nutricia Ltd., UK) added to placebo drink<br>Placebo 250 mL of sugar-free lemon squash (Tesco,UK) and water;*f* = 2 doses<br>Study 2: 150 mg/kg tyrosine assessed further; supplements as described above; *f* = Single dose	Study 1:Determined optimal tyrosine dose (no cog measures)<br>Study 2: Exercise in a hot environment	Study 2: Simple reaction time, attention (vigilance test, dual-task test)	Study 2: All cognitive measures: NS
Jongkees et al. (2017)	Double-blind , between-subjects design (follow-on from Colzato 2013);counterbalanced	Study 1:AssessTyrosine on working memory	n = 36; aged 18-30 y (Tyrosine 22.2, 2.4; Placebo 20.8, 1.9); 2 Males; 34 Females	2 g tyrosine (Bulk Powders Ltd.) or placebo 2 g microcrystalline cellulose (Sigma-Aldrich) ; *f* = single dose (dissolved in 400ml of orange juice)	No moderator	Verbal Working memory (N-back task: 2-back and 3-back)	Effect on WM load (p = .003);main effect of Tyr (p = .013) on target sensitivity (improved); Hits and misses tyr positive effect [$F(1,330 = 5.30$, $p = .028$. partial $\eta^2 = .138$;correct rejections and false alarms [$F(1, 33) = 6.81$, $p = .14$, partial $\eta^2 = .171$]
Kishore et al. (2013)	Randomised, double-blind,	Assess the effect of tyrosine as a	n = 10; aged 20-30 y (ND,ND); 10 Males	100 mg/kg tyrosine 50 g low fat, high-energy	Heat stress	Event related potentials (P300, CNV)	P300 latency: heat exposure (HE) $p < .01$;

	placebo-controlled, crossover design; counterbalanced; 1-week washout	countermeasure in the reduction of information processing and cognitive decline following heat stress	(Indian Army); 0 Females	bar (containing 6.5 g of L-tyrosine) Placebo 50 g low fat, high-energy bar; *f* = Single dose			CNV (M100 latency): HE $p < .05$; (M200 latency): NS; (M100 amplitude): HE increased for tyrosine, $p < .05$
Magill et al. (2003) #	Randomised, double-blind, placebo-controlled, independent groups design	Compare the effects of placebo versus D-amphetamine, caffeine, tyrosine and phentermine on cognitive and motor performance during long-term sleep deprivation	n = 76; aged 18-35 y (ND, ND); 76 Males; 0 Females	150 mg/kg of tyrosine or placebo	Long-term sleep deprivation	Attention (visual scanning, visual vigilance, four-choice serial reaction time), memory (long-term memory), working memory (running memory task), executive function (logical reasoning, Stroop); mathematical processing, time wall task, pursuit tracking task, trails (B) task	Running memory RT: $p < .012$; logical reasoning RT: $p < .012$; vigilance RT: $p < .006$; vigilance hits: $p < .001$; Stroop RT: $p < .001$; 4-choice RT: $p < .042$ at 5.5h; mathematical processing RT: $p < .003$ at 1.5h;Running memory, logical reasoning, mathematical processing, visual vigilance deteriorated with sleep dep., $p \leq .021$
Mahoney et al. (2007) #	Double-blind, placebo-controlled, crossover design; counterbalanced; 3-day washout	Determine whether severe cold exposure impairs cognition and whether tyrosine can mitigate such deficits	n = 19; aged (20.5, ND); Males; Females: ND	150 mg/kg tyrosine or placebo (energy bar) prior to each water immersion (thermoneutral or cold); *f* = 300 mg/kg (2 bars)	Severe cold exposure	Attention (visual vigilance, four-choice visual RT), working memory (delayed match-to-sample)	Match-to-sample (correct responses: $p < .05$; RT: $p < .05$ at 1st immersion, NS at 2nd); four-choice RT (treatment: NS); visual vigilance: NS
O'Brien et al. (2007) #	Double-blind, placebo-controlled, crossover design; counterbalanced;	Evaluate the effect of tyrosine on cognitive, physical and psychomotor performance during	n = 15; aged (20, ND); 14 Males (enlisted soldiers); 0 Females	150 mg/kg tyrosine or placebo (energy bar) prior to each water immersion (thermoneutral or	Cold-air exposure after cold water immersions	Attention (visual vigilance, four-choice visual RT), working memory (delayed match-to-sample, serial	Match-to-sample: (placebo reduced % correct) $p = .023$; addition/subtraction (RT placebo slower): $p =$

	1-week washout	cold-air exposure after cold water immersions		cold); *f* = 300 mg/kg (2 bars)		addition/subtraction task, repeated acquisitions), executive function (logical reasoning), marksmanship	.0084; vigilance (placebo & tyrosine improved): p = .01<br>- All other cognitive measures: NS
Steenbergen et al. (2015)	Randomised, double-blind, placebo-controlled, crossover design; counterbalanced; 3 to 7-day washout	Assess the effect of tyrosine on cognitive flexibility (as measured by task-switching)	n = 22; aged (19.3, 1.5); 0 Males; 22 Females	2 g tyrosine (Bulk Powders Ltd.) or Placebo 2 g microcrystalline cellulose (Sigma-Aldrich Co. LLC); *f* = single dose (dissolved in 400ml of orange juice)	Cognitive flexibility	Cognitive flexibility (response-stimulus interval: short vs long RSI; task repetition: repetition vs alternation)	Long RSI: p = .009, short RSI: NS; repetition: NS tyrosine reduced switching costs but only for long RSI
Watson et al. (2012)	Randomised, single-blind, placebo-controlled, crossover design; counterbalanced; 1-week washout	Examine the effect of tyrosine supplementation on prolonged exercise capacity and cognitive function during exercise in a warm environment	n = 8; aged (23,3); 8 Males; 0 Females	150 mg/kg tyrosine (SHS Intl., Liverpool, UK) in a sugar-free fruit drink (Tesco Ltd., Chestnut, UK) or placebo sugar-free fruit drink (Tesco Ltd., Chestnut, UK); *f* = 2 doses	Warm environment	Attention (RVIP), memory (Stenberg memory-scanning task), executive function (Stroop)	All cognitive measures: NS
B Vitamins	(n = 2 studies)						
Bryan et al. (2002)	Randomised, double-blind, placebo-controlled,	Investigated folate, vitamin B_6 and B_{12} supplementation on cognitive function in healthy women	n = 56; aged 20-30 y (25.2,3.2); 0 Males; 56 Females	750 µg folate, 15µg B_{12}, 75 mg B_6 or placebo; *f* = single dose (1 capsule;75%		Information processing (boxes test, digit symbol-coding, symbol search), working memory (digit span-backwards, letter-	All cognitive measures: NS

	independent groups design	from three different age groups.		of tolerable daily upper limit), 5-weeks		number sequencing), memory (RAVLT, recall of symbols, activity recall), executive function (Stroop, self-ordered pointing task, uses for objects, trail making test, verbal fluency), verbal ability (vocabulary, spot-the-word)	
Systematic Literature Reviews	(n = 7 manuscripts)						
Attipoe et al. (2015)		Systematic literature review on Tyrosine					
Crawford et al (2017)		Systematic literature review on Caffeine					
Geng et al (2010)		Systematic literature review on Ginseng					
Hoyland et al. (2008)		Systematic literature review on Macronutrients					
Ko et al. (2014)		Systematic literature review on β-alanine					

Neale et al. (2012)	Systematic literature review on Ginseng
Teo et al. (2017)	Systematic literature review on Omega-3

Abbreviations and symbols:# Studies/manuscripts that have been scrutinised and quality scored by the aforementioned respective supplement Systematic Literature reviews ANT, attention network task; SDlat, Latency SD SDS, speed maintenance score; JDS, Johns Drowsiness Scale; RNG, random number generation; SVRT, simple visual reaction time; RVRT, recognition visual reaction time; PVT, psychomotor vigilance test; FVT, field vigilance test; AE, ??; VRT, visual reaction time; RT, reaction time; JEF, Jansari assessment of executive function; CBF, cerebral blood flow; DSST, digit symbol substitution test; CF, cocoa flavanols; RVIP , rapid visual information processing; EGCG, epigallocatechin gallate; PRM, pattern recognition memory; SRM, spatial recognition memory; WM, working memory; QoM, quality of memory; SM, secondary memory; SoM , speed of memory; SoA, speed of attention; QoA, quality of attention; AoA, accuracy of attention; CDB, Cognitive Demand Battery; ERP, event related potentials; DHA, docosahexaenoic acid; EPA, eicosapentaenoic acid; RAVLT, Rey Auditory Verbal Learning Test; SCWT, Stroop colour and word test; TMT, trail making test; RAT, remote associates task; AUT, alternate uses task

Table S3. Excluded papers and reason for exclusion

**Supplement / Area of interest** (# papers)	**Papers** (Author; Year)	**Experiment / Review** (E / R)	**Excluded** (level of exclusion)
**Supplements**			
**Ampakine** (1)			
	Porrino et al. (2005)	E	Yes – abstract
**Bacopa** (13)			
	Benson et al. (2014)	E	Yes – abstract
	Ko et al. (2014)	R	Yes – abstract
	Kongkeaw et al. (2014)	R	Yes – after full review
	Nathan et al. (2001)	E	Yes – abstract
	Pase et al. (2012)	R	Yes – after full review
	Roodenrys et al. (2002)	E	Yes – abstract
	Sathyanarayanan et al. (2012)	E	Yes – abstract
	Solis et al. (2015)	E	Yes – abstract
	Stough et al. (2001)	E	Yes – abstract
	Stough et al. (2008)	E	Yes – abstract
	Stough et al. (2008)	E	Yes – duplicate
	Stough et al. (2013)	R	Yes – after full review
	Trexler et al. (2015)	R	Yes – abstract
**Beta-alanine** (4)			
	Hoffman et al. (2014)	E	Yes – duplicate
	Ko et al. (2014)	R	Yes – abstract
	Solis et al. (2015)	E	Yes – abstract
	Trexler et al. (2015)	R	Yes – abstract
**Caffeine** (34)			
	Aidman et al. (under review)	E	Yes – duplicate
	Borota et al. (2014)	E	Yes – after full review
	Cappelletti et al. (2015)	R	Yes – abstract
	Doan et al. (2006)	E	Yes – abstract
	Glade (2010)	R	Yes – after full review
	Goldstein et al. (2010)	R	Yes – after full review
	Goldstein et al. (2010)	R	Yes – duplicate
	Hamleers et al. (2000)	E	Yes – abstract
	Harvanko et al. (2015)	E	Yes – abstract
	Heatherley et al. (2005)	E	Yes – abstract
	Heilbronner et al. (2015)	E	Yes – abstract
	Hogevorst et al. (2008)	E	Yes – after full review
	Kennedy & Scholey (2004)	E	Yes – abstract
	Killgore et al. (2009)	E	Yes – abstract
	Killgore et al. (2014)	E	Yes – after full review
	Lanini et al. (2016)	E	Yes – abstract
	Mahoney et al. (2012)	E	Yes – abstract
	Maridakis et al. (2009a)	E	Yes – after full review
	Maridakis et al. (2009b)	E	Yes – after full review
	McClung et al. (2011)	E	Yes – after full review
	McLellan et al. (2016)	R	Yes – after full review
	Nehlig (2010)	R	Yes – after full review
	Paulus et al. (2015)	E	Yes – abstract
	Pray et al. (ND)	-	Yes – abstract
	Renda et al. (2015)	E	Yes – abstract

	Rettner (2013)	-	Yes – abstract
	Smith (2002)	R	Yes – after full review
	Smith (2009)	E	Yes – after full review
	Smith et al. (2005)	E	Yes – abstract
	Sherman et al. (2016)	E	Yes – after full review
	Ullrich et al. (2015)	E	Yes – abstract
	Warburton et al. (2001)	E	Yes – abstract
	Wesensten et al. (2002)	E	Yes – after full review
	Wesensten et al. (2004)	E	Yes – abstract
**Curcumin** (1)			
	Davis et al. (2007)	E	Yes – abstract
**DHEA** (7)			
	Mendoza et al. (2016)	E	Yes – abstract
	Shia et al. (2015)	E	Yes – abstract
	Shield et al. (2016)	E	Yes – abstract
	Taylor et al. (2016)	E	Yes – abstract
	Vale et al. (2015)	E	Yes – abstract
	Vale et al. (2016)	E	Yes – abstract
	Wang et al. (2016)	E	Yes – abstract
**Flavanoids** (16)			
	Alharbi et al. (2015)	E	Yes – abstract
	Bell et al. (2015)	R	Yes – after full review
	Bell et al. (2015)	R	Yes – duplicate
	Bondonno et al. (2014)	E	Yes – abstract
	Caldwell et al. (2015)	E	Yes – after full review
	Camfield et al. (2014)	R	Yes – after full review
	Cermak et al. (2012)	E	Yes – abstract
	Field et al. (2011)	E	Yes – after full review
	Hendrickson & Mattes (2008)	E	Yes – abstract
	Pase et al. (2013)	E	Yes – abstract
	Saunders & Spencer (2012)	R	Yes – abstract
	Scholey et al. (2012)	E	Yes – abstract
	Smeriglio et al. (2016)	R	Yes – abstract
	Thorp et al. (2009)	E	Yes – abstract
	Whyte & Williams (2015)	E	Yes – abstract
	Whyte et al. (2015)	E	Yes – abstract
**Gingko** (10)			
	Burns et al. (2005)	E	Yes – abstract
	Cieza et al. (2003)	E	Yes – abstract
	Hartley et al. (2003)	E	Yes – abstract
	Kennedy et al. (2001)	E	Yes – abstract
	Kennedy et al. (2007)	E (x3)	Yes – after full review
	Mix et al. (2000)	E	Yes – abstract
	Nathan et al. (2002)	E	Yes – abstract
	Santos et al. (2003)	E	Yes – abstract
	Snitz et al. (2009)	E	Yes – abstract
	Soloman et al. (2002)	E	Yes – abstract
**Ginseng** (4)			
	Kennedy et al. (2007)	E	Yes – abstract
	Oliynyk & Oh (2013)	R	Yes – after full review
	Scholey et al. (2010)	E	Yes – abstract
	Shergis et al. (2013)	R	Yes – abstract
**Glucose and macro.** (4)			
	Hoyland et al. (2008)	R	Yes – abstract
	Lamport et al. (2009)	R	Yes – abstract
	Smith et al. (2010)	R	Yes – after full review
	Sunram-Lea et al. (2008)	E	Yes – after full review

**Guarana / Guarana+Multivitamin** (2)			
	Scholey et al. (2013)	E	Yes – abstract
	Veasey et al. (2015)	E	Yes – duplicate
**Iron** (3)			
	Cairns et al. (2005)	E	Yes – abstract
	Mielgo-Ayuso et al. (2015)	E	Yes – abstract
	Wilson & Brothers (2010)	R	Yes – abstract
**Meldonium** (1)			
	Stuart et al. (2016)	E	Yes – abstract
**Micronutrients** (5)			
	Celep et al. (2017)	-	Yes – abstract
	Chellappa & Karunanidhi (2012)	E	Yes – abstract
	Huskisson et al. (2007)	R	Yes – after full review
	Newton (2009)	E	Yes – abstract
	Potgieter (2013)	R	Yes – abstract
**Modafinil** (2)			
	Battleday & Brem (2015)	R	Yes – abstract
	European College of Neuropsychopharmacology (ND)	-	Yes – abstract
**Multivitamins** (10)			
	Camfield et al. (2013)	E	Yes – abstract
	Camfield et al. (2013)	E	Yes – duplicate
	Harris et al. (2015)	E	Yes – abstract
	Harris et al. (2015)	E	Yes – duplicate
	Haskell et al. (2010)	E	Yes – abstract
	Kennedy et al. (2016)	E	Yes – abstract
	Kennedy et al. (2016)	E	Yes – duplicate
	Pipingas et al. (2014)	E	Yes – abstract
	White et al. (2015)	E	Yes – abstract
	White et al. (2015)	E	Yes – duplicate
**Nigella Sativa** (1)			
	Sayeed et al. (2013)	E	Yes – abstract
**Nitrate** (4)			
	Baker et al. (2016)	R	Yes – after full review
	Baker et al. (2016)	R	Yes – duplicate (DST report)
	Bondonno et al. (2014)	E	Yes – duplicate
	Kelly et al. (2013)	E	Yes – abstract
**Omega 3** (54)			
	Aid et al. (2005)	E	Yes – abstract
	Albert et al. (2016)	R	Yes – abstract
	Aluko (2012)	-	Yes – abstract
	Bailes & Patel (2014)	R	Yes – after full review
	Bailes & Patel (2014)	R	Yes – duplicate
	Bauer et al. (2014)	R	Yes – after full review
	Bauer et al. (2014)	R	Yes – duplicate
	Brown Evidence-based Practice Centre (2016)	-	Yes – abstract
	Carter et al. (2013)	E	Yes – abstract
	Cornum (2014)	-	Yes – abstract
	Cornum (2014)	-	Yes – duplicate
	Coulter (2014)	-	Yes – abstract
	Coulter (2014)	-	Yes – duplicate
	Dangour et al. (2010)	E	Yes – abstract
	Davis & Prall (2014)	-	Yes – abstract
	Deuster (2014)	-	Yes – abstract
	Dyall (2011)	R	Yes – after full review

	Fontani et al. (2005a)	E	Yes – abstract
	Fontani et al. (2005a)	E	Yes – duplicate
	Fontani et al. (2005b)	E	Yes – after full review
	Fontani et al. (2009)	E	Yes – abstract
	Giles et al. (2014)	R	Yes – after full review
	Grosso et al. (2014)	R	Yes – abstract
	Hamazaki et al. (2014)	R	Yes – after full review
	Hibbeln & Gow (2014)	R	Yes – abstract
	Howe & Buckley (2014)	R	Yes – abstract
	Jackson et al. (2012c)	E	Yes – abstract
	Jackson et al. (2015)	E	Yes – abstract
	Johnsons (2011)	E	Yes – after full review
	Johnsons (2011)	E	Yes – duplicate
	Johnston et al. (2013)	E	Yes – abstract
	Kidd (2007)	R	Yes – after full review
	Kim (2015)	-	Yes – abstract
	Lands (2014)	-	Yes – abstract
	Lewis et al. (2011)	R	Yes – abstract
	Marriott et al. (2014)	E	Yes – abstract
	McCarthy et al. (2014)	R	Yes – abstract
	Mickleborough (2013)	E	Yes – abstract
	Military Medicine (journal) (2014)	-	Yes – abstract
	Montain & Jonas (2014)	-	Yes – abstract
	Montain & Jonas (2014)	-	Yes – duplicate
	Montgomery et al. (2013)	E	Yes – abstract
	Muldoon et al. (2014)	R	Yes – after full review
	Muldoon et al. (2014)	R	Yes – duplicate
	Newberry et al. (2015)	R	Yes – abstract
	Nokdhes & Sittiprapaporn (2017)	E	Yes – abstract
	Paajanen (2007)	E	Yes – abstract
	Radcliffe et al. (2016)	R	Yes – after full review
	Shei et al. (2014)	-	Yes – abstract
	Stonehouse et al. (2013)	E	Yes – abstract
	Strike et al. (2016)	E	Yes – abstract
	van der Wurff et al. (2016)	E	Yes – abstract
	Wu et al. (2008)	E	Yes – abstract
	Xiao et al. (2013)	E	Yes – abstract
**Polyphenol** (2)			
	Zhao et al. (2016)	E	Yes – abstract
	Zhao et al. (2016)	E	Yes – duplicate
**Prebiotics & Probiotics** (50)			
	Ahire et al. (2011)	E	Yes – abstract
	Allen et al. (2016)	E	Yes – abstract
	Allen et al. (2016)	E	Yes – duplicate
	Allen et al. (2017)	R	Yes – after full review
	Benton et al. (2007)	E	Yes – abstract
	Burokas et al. (2015)	R	Yes – abstract
	Callaghan et al. (2016)	E	Yes – abstract
	Carabotti et al. (2015)	R	Yes – abstract
	Carabotti et al. (2015)	R	Yes – duplicate
	Chung et al. (2014)	E	Yes – abstract
	Cowan et al. (2016)	E	Yes – abstract
	Desbonnet et al. (2015)	E	Yes – abstract
	Desbonnet et al. (2008)	E	Yes – abstract
	Diamond et al. (2011)	R	Yes – abstract
	Dinan et al. (2013)	R	Yes – abstract
	Dinan et al. (2015)	R	Yes – abstract

	Dinan et al. (2015)	R	Yes – duplicate
	Dinan et al. (2015)	R	Yes – duplicate
	Dinan et al. (2015)	R	Yes – duplicate
	Forsythe et al. (2010)	R	Yes – abstract
	Foster & McVey Neufeld (2013)	R	Yes – abstract
	Foster (2016)	R	Yes – abstract
	Friedrich (2015)	R	Yes – after full review
	Jiang et al. (2015)	E	Yes – abstract
	Leung & Thuret (2015)	R	Yes – abstract
	Liang et al. (2015)	E	Yes – abstract
	Liang et al. (2015)	E	Yes – duplicate
	Liu et al. (2016)	E	Yes – abstract
	Logan & Katzman (2005)	R	Yes – abstract
	Logan & Katzman (2005)	R	Yes – duplicate
	Luna & Foster (2015)	R	Yes – abstract
	Lyte (2011)	-	Yes – abstract
	Mayer et al. (2013)	R	Yes – after full review
	Mayer et al. (2013)	R	Yes – duplicate
	Mayer et al. (2014)	R	Yes – abstract
	Messaoudi et al. (2011)	E	Yes – abstract
	Messaoudi et al. (2011)	E	Yes – abstract
	Monteil-Castro et al. (2013)	R	Yes – abstract
	Monteil-Castro et al. (2013)	R	Yes – duplicate
	Moore (2011)	R	Yes – abstract
	Patterson et al. (2014)	-	Yes – abstract
	Pyne et al. (2014)	R	Yes – abstract
	Saxelin (2008)	R	Yes – abstract
	Schmidt et al. (2015)	E	Yes – abstract
	Scholz-Ahrens et al. (2007)	R	Yes – abstract
	Sheridan et al. (2014)	R	Yes – abstract
	Smith (2005)	E	Yes – abstract
	Steenbergen et al. (2015)	E	Yes – abstract
	Tillisch et al. (2013)	E	Yes – after full review
	Yeon et al. (2010)	E	Yes – abstract
**Protein** (2)			
	Ferrando (2013)	R	Yes – abstract
	Pasiakos et al. (2015)	-	Yes – abstract
**Tryptophan** (4)			
	Jenkins et al. (2016)	R	Yes – after full review
	Jenkins et al. (2016)	R	Yes – duplicate
	Mohajeri et al. (2015)	E	Yes – abstract
	Booij et al. (2006)	E	Yes – abstract
**Tyrosine** (7)			
	Attipoe et al. (2015)	R	Yes – abstract
	Hase et al. (2015)	R	Yes – after full review
	Jongkees et al. (2015)	R	Yes – after full review
	Chinevere et al. (2002)	E	Yes – abstract
	Palinkas et al. (2007)	E	Yes – abstract
	Sutton et al. (2005)	E	Yes – abstract
	Tumilty et al. (2014)	E	Yes – abstract
**Vitamin B** (3)			
	Huskisson et al. (2007)	R	Yes – after full review
	Kennedy (2016)	R	Yes – after full review
	Kennedy (2016)	R	Yes – duplicate
**Vitamin D** (11)			
	Annweiler et al. (2009)	R	Yes – abstract
	Buell & Dawson-Hughes (2008)	R	Yes – abstract

	Colon (2016)	-	Yes – after full review
	Dalquist et al. (2015)	R	Yes – abstract
	Gertner & Horn (2008)	R	Yes – abstract
	Gertner & Horn (2008)	R	Yes – duplicate
	Hiserote et al. (2016)	E	Yes – abstract
	Lutz et al. (2012)	E	Yes – abstract
	Przybelski & Binkley (2007)	E	Yes – abstract
	Wentz et al. (2014)	R	Yes – after full review
	Zittermann & Koerfer (2008)	R	Yes – abstract
**Vitamin E** (1)			
	Klein et al. (2011)	E	Yes – abstract
**Zinc** (3)			
	Sawada & Yokoi (2010)	E	Yes – abstract
	Takeda & Tamano (2016)	R	Yes – abstract
	Takeda et al. (2015)	E	Yes – abstract
**Defence Interests**			
**Background** (7)			
	Canadian Forces Health Services Group (ND)	-	Yes – abstract
	Forbes et al. (2015)	R	Yes – abstract
	Kime (2016)	-	Yes – abstract
	Kuszak et al. (2016)	R	Yes – abstract
	Smith et al. (2016)	-	Yes – abstract
	Williams et al. (2008)	-	Yes – abstract
	Williams et al. (2008)	-	Yes – duplicate
**Climate considerations** (3)			
	Paulus et al. (2009)	R	Yes – after full review
	Paulus et al. (2009)	R	Yes – duplicate
	Taylor et al. (2016)	R	Yes – after full review
**DSTG Review** (2)			
	"Impacts on performance" word doc	R	Yes – after full review
	"Impacts on performance" word doc	R	Yes - duplicate
**Ethical considerations** (3)			
	Jacobs et al. (ND)	R	Yes – after full review
	Jacobs et al. (ND)	R	Yes – duplicate
	Sauter & Gerlinger (2013)	R	Yes – after full review
**General review** (1)			
	Gomez-Pinilla (presentation)	-	Yes – abstract
**Military use** (22)			
	Austin et al. (2015)	E	Yes – abstract
	Austin et al. (2015)	E	Yes – duplicate
	Bovill et al. (2002)	E	Yes – abstract
	Brink (2007)	-	Yes – abstract
	Casey et al. (2014)	E	Yes – abstract
	Casey et al. (2014)	E	Yes – duplicate
	Coulter et al. (2011)	-	Yes – abstract
	Deuster & Simmons (2004)	-	Yes – after considerable review
	Goertz et al. (2013)	E	Yes – abstract
	Greenwood & Oria (2008)	-	Yes – after considerable review
	Hoedebecke (2015)	R	Yes – abstract
	Kjertakov et al. 2013	E	Yes – abstract
	Knapik et al. (2014)	R	Yes – abstract
	Knapik et al. (2014)	R	Yes – duplicate
	Knapik et al. (2016a)	E	Yes – abstract

	Knapik et al. (2016b)	-	Yes – abstract
	Lieberman (2013)	-	Yes – abstract
	Lieberman et al. (2010)	E	Yes – abstract
	McClung et al. (2016)	R	Yes – abstract
	Morioka et al. (2015)	E	Yes – abstract
	Smith et al. (2013)	E	Yes – abstract
	Tomczynska-Mleko et al. (2013)	E	Yes – abstract
**Nutrition/Diet** (6)			
	Beilharz et al. (2015)	R	Yes – abstract
	Beilharz et al. (2015)	R	Yes – duplicate
	Gorby et al. (2010)	R	Yes – after full review
	Jenkins et al. (2016)	R	Yes – duplicate
	Mellendijk et al. (2015)	R	Yes – abstract
	Smith et al. (2015)	E	Yes – duplicate
**Nutritional fitness test** (3)			
	Florez et al. (2014)	R	Yes – after full review
	Florez et al. (2014)	R	Yes – duplicate
	Lieberman (2003)	R	Yes – after full review
**Performance Enhancing** (5)			
	Cohen et al. (2016)	E	Yes – abstract
	Kelley et al. (2010)	-	Yes – after full review
	Landau et al. (2012)	E	Yes – abstract
	Lynch et al. (2014)	R	Yes – abstract
	Manza et al. (2016)	E	Yes – abstract
**Resilience** (4)			
	Florez et al. (2014)	R	Yes – duplicate
	Florez et al. (2014)	R	Yes – duplicate
	Hancock & Warm (2012)	-	Yes – abstract
	Pattyn et al. (2014)	E	Yes – abstract
**Safety** (18)			
	Arensberg et al. (2014)	-	Yes – after full review
	Austin et al. (2016)	E	Yes – after full review
	Boos et al. (2010)	-	Yes – abstract
	Carvey et al. (2012)	E	Yes – abstract
	Deuster & Lieberman (2016)	R	Yes – abstract
	Deuster & Lieberman (2016)	R	Yes – duplicate
	Eliason et al. (2012)	-	Yes – abstract
	Gijsbert et al. (2008)	R	Yes – after full review
	Kime (2016)	-	Yes – duplicate
	Knapik (2016a)	E	Yes – duplicate
	Lieberman et al. (2010)	E	Yes – duplicate
	Rettner (2013)	-	Yes – duplicate
	Stranger et al. (2012)	R	Yes – after full review
	Urban & Gao (2014)	R	Yes – abstract
	Urban & Gao (2014)	R	Yes – duplicate

Section/topic	#	Checklist item	Reported on page #
**TITLE**			
Title	1	Identify the report as a systematic review, meta-analysis, or both.	1
**ABSTRACT**			
Structured summary	2	Provide a structured summary including, as applicable: background; objectives; data sources; study eligibility criteria, participants, and interventions; study appraisal and synthesis methods; results; limitations; conclusions and implications of key findings; systematic review registration number.	2
**INTRODUCTION**			
Rationale	3	Describe the rationale for the review in the context of what is already known.	3-5
Objectives	4	Provide an explicit statement of questions being addressed with reference to participants, interventions, comparisons, outcomes, and study design (PICOS).	5,6,41
**METHODS**			
Protocol and registration	5	Indicate if a review protocol exists, if and where it can be accessed (e.g., Web address), and, if available, provide registration information including registration number.	1,5
Eligibility criteria	6	Specify study characteristics (e.g., PICOS, length of follow-up) and report characteristics (e.g., years considered, language, publication status) used as criteria for eligibility, giving rationale.	5,6
Information sources	7	Describe all information sources (e.g., databases with dates of coverage, contact with study authors to identify additional studies) in the search and date last searched.	6,7
Search	8	Present full electronic search strategy for at least one database, including any limits used, such that it could be repeated.	TableS1
Study selection	9	State the process for selecting studies (i.e., screening, eligibility, included in systematic review, and, if applicable, included in the meta-analysis).	6,7,8
Data collection process	10	Describe method of data extraction from reports (e.g., piloted forms, independently, in duplicate) and any processes for obtaining and confirming data from investigators.	7,8
Data items	11	List and define all variables for which data were sought (e.g., PICOS, funding sources) and any assumptions and simplifications made.	6,41

Risk of bias in individual studies	12	Describe methods used for assessing risk of bias of individual studies (including specification of whether this was done at the study or outcome level), and how this information is to be used in any data synthesis.	8
Summary measures	13	State the principal summary measures (e.g., risk ratio, difference in means).	NA
Synthesis of results	14	Describe the methods of handling data and combining results of studies, if done, including measures of consistency (e.g., I^2) for each meta-analysis.	NA

Section/topic	#	Checklist item	Reported on page #
Risk of bias across studies	15	Specify any assessment of risk of bias that may affect the cumulative evidence (e.g., publication bias, selective reporting within studies).	8
Additional analyses	16	Describe methods of additional analyses (e.g., sensitivity or subgroup analyses, meta-regression), if done, indicating which were pre-specified.	NA
**RESULTS**			
Study selection	17	Give numbers of studies screened, assessed for eligibility, and included in the review, with reasons for exclusions at each stage, ideally with a flow diagram.	Figure1,Table2,TableS4, Table S5
Study characteristics	18	For each study, present characteristics for which data were extracted (e.g., study size, PICOS, follow-up period) and provide the citations.	8,9
Risk of bias within studies	19	Present data on risk of bias of each study and, if available, any outcome level assessment (see item 12).	TableS6
Results of individual studies	20	For all outcomes considered (benefits or harms), present, for each study: (a) simple summary data for each intervention group (b) effect estimates and confidence intervals, ideally with a forest plot.	10-27,Table2,Table3
Synthesis of results	21	Present results of each meta-analysis done, including confidence intervals and measures of consistency.	NA
Risk of bias across studies	22	Present results of any assessment of risk of bias across studies (see Item 15).	Table S6
Additional analysis	23	Give results of additional analyses, if done (e.g., sensitivity or subgroup analyses, meta-regression [see Item 16]).	NA
**DISCUSSION**			
Summary of evidence	24	Summarize the main findings including the strength of evidence for each main outcome; consider their relevance to key groups (e.g., healthcare providers, users, and policy makers).	27-29

Limitations	25	Discuss limitations at study and outcome level (e.g., risk of bias), and at review-level (e.g., incomplete retrieval of identified research, reporting bias).	2-31
Conclusions	26	Provide a general interpretation of the results in the context of other evidence, and implications for future research.	31-33,TableS2,TableS3
**FUNDING**			
Funding	27	Describe sources of funding for the systematic review and other support (e.g., supply of data); role of funders for the systematic review.	33

From: Moher D, Liberati A, Tetzlaff J, Altman DG, The PRISMA Group (2009). Preferred Reporting Items for Systematic Reviews and Meta-Analyses: The PRISMA Statement. PLoS Med 6(7): e1000097. doi:10.1371/journal.pmed1000097

For more information, visit: **www.prisma-statement.org**.

Table S5. GRADE analysis: Overall evidence synthesis for the impact of reviewed dietary supplements on cognitive domains.

Dietary supplement	Moderator & outcome	Total participants (studies)	Confidence in effect estimate[a]	Safety[b]	GRADE recommendation[c]
**Beta alanine**	**Fatigue**				
	Psychomotor	20(1)	C	+1	No recommendation
**Tyrosine**	**Sleep Deprivation**				
	Psychomotor	109(2)	C	+2	Conditional
	Vigilance	76(1)	C	+2	No recommendation
	Memory	109(2)	B	+2	Conditional
	Maths	76(1)	D	+2	No recommendation
	Logical reasoning	76(1)	C	+2	No recommendation
	**Noise**				
	Psychomotor	20(1)	C	+2	No recommendation
	Info processing speed	26(2)	C	+2	No recommendation
	Vigilance	36(2)	C	+2	No recommendation
	Memory	36(2)	C	+2	No recommendation
	**Climate**				
	Psychomotor	23(2)	C	+2	No recommendation
	Info processing speed	44(3)	C	+2	No recommendation
	Attention	27(2)	C	+2	No recommendation
	Vigilance	19(1)	C	+2	No recommendation
	Memory	8(1)	C	+2	No recommendation
	Maths	15(1)	C	+2	No recommendation
	WM	19(1)	C	+2	No recommendation
	Logical reasoning	15(1)	C	+2	No recommendation
	**Altitude & Climate**				
	Complex RT	23(1)	C	+2	No recommendation
	Vigilance	23(1)	C	+2	No recommendation
	Memory	23(1)	C	+2	No recommendation
	Map reading	23(1)	C	+2	No recommendation
	Maths	23(1)	C	+2	No recommendation
	**Exercise & Heat**				
	Attention	16(2)	C	+2	No recommendation
	Vigilance	16(2)	C	+2	No Recommendation
	**Lower Body Negative Pressure**				
	Complex RT	22(1)	C	+2	No recommendation
	Vigilance	22(1)	C	+2	No recommendation
	**No Stressor**				
	Psychomotor	20(1)	C	+2	No recommendation
	Simple RT	20(1)	C	+2	No recommendation
	Complex RT	20(1)	C	+2	No recommendation
	WM	58(2)	C	+2	No recommendation
	Cognitive flexibility	54(2)	C	+2	No recommendation

	Inhibitory control	22(1)	C	+2	No recommendation
**Omega-3**	**None**				
	Simple RT	524(4)	B	+2	No recommendation
	Complex RT	524(4)	B	+2	No recommendation
	Attention	140(1)	C	+2	No recommendation
	Vigilance	65(1)	C	+2	No recommendation
	Sustained attention	490(3)	B	+2	No recommendation
	Memory	520(4)	B	+2	No recommendation
	WM	218(2)	C	+2	No recommendation
	Attentional Control	72(1)	C	+2	No recommendation
	Inhibitory control	261(4)	B	+2	No recommendation
	Logical reasoning	54(1)	C	+2	No recommendation
	Cognitive flexibility	41(1)	C	+2	No recommendation
**Vitamin B1**	**None**				
	Info processing speed	56(1)	C	+2	No recommendation
	Memory	56(1)	C	+2	No recommendation
	WM	56(1)	C	+2	No recommendation
	Inhibitory control	56(1)	C	+2	No recommendation
	Cognitive flexibility	56(1)	C	+2	No recommendation
**Nitrate**	**Cognitive & Physical Fatigue**				No recommendation
	Attention	16(1)	C	+2	No recommendation
	Inhibitory control	16(1)	C	+2	No recommendation
	**None**				
	Info processing speed	40(1)	C	+2	No recommendation
	WM	40(1)	C	+2	No recommendation
**Caffeine**	**Sleep Deprivation**				
	Psychomotor	51(3)	C	+2	No recommendation
	Simple RT	10(1)	C	+2	No recommendation
	Complex RT	78(3)	B	+2	No recommendation
	Attention	393(14)	B	+2	Conditional
	Vigilance	413(15)	B	+2	No recommendation
	Memory	178(5)	B	+1	Conditional
	Problem solving	159(6)	B	+2	Conditional
	Logical reasoning	179(7)	B	+2	Conditional
	**Mental exertion**				
	Complex RT	24(1)	C	+2	No recommendation
	Problem solving	17(1)	C	+2	No recommendation
	Logical reasoning	17(1)	C	+2	No recommendation
	**Physical Exertion**				
	Simple RT	17(1)	C	+2	No recommendation
	Attention	17(1)	C	+2	No recommendation
	Vigilance	17(1)	C	+2	No recommendation
	Memory	17(1)	C	+2	No recommendation
	**Sleep Deprivation & Sustained Operations**				

	Attention	96(3)	C	+2	Conditional
	Vigilance	96(3)	C	+2	Conditional
	Memory	76(1)	C	+2	No recommendation
	**Physical exertion & climate**				
	Complex RT	9(1)	C	+2	No recommendation
	**None**				
	Info processing speed	43(1)	C	+2	No recommendation
	Simple RT	20(1)	C	+2	No recommendation
	Complex RT	63(2)	C	+2	No recommendation
	Attention	36(1)	C	+2	No recommendation
	Vigilance	36(1)	C	+2	No recommendation
	Memory	69(2)	C	+2	No recommendation
	Inhibitory control	36(1)	C	+2	No recommendation
	Cognitive flexibility	43(1)	C	+2	No recommendation
	Planning	43(1)	C	+2	No recommendation
**Flavanoids**	**None**				
	Info processing speed	30(1)	D	+2	No recommendation
	Simple RT	63(2)	D	+2	No recommendation
	Attention	63(2)	D	+2	No recommendation
	Vigilance	36(1)	C	+2	No recommendation
	Memory	28(1)	D	+2	No recommendation
	Sustained Attention	66(2)	D	+2	No recommendation
	WM	85(3)	C	+2	No recommendation
	Inhibitory control	81(3)	D	+2	No recommendation
	Cognitive flexibility	28(1)	D	+2	No recommendation
	Logical reasoning	64(2)	D	+2	No recommendation
**Gingko biloba**	**None**				
	Info processing speed	128(4)	C	0	No recommendation
	Simple RT	60(1)	C	0	No recommendation
	Attention	140(4)	C	0	No recommendation
	Memory	220(5)	C	0	No recommendation
	WM	160(5)	C	0	No recommendation
	Cognitive flexibility	92(2)	C	0	No recommendation
**Ginseng**	**None**				
	Info processing speed	170(6)	D	+2	No recommendation
	Simple RT	47(2)	D	+2	No recommendation
	Complex RT	60(2)	D	+2	No recommendation
	Attention	133(5)	D	+2	No recommendation
	Vigilance	15(1)	D	+2	No recommendation
	Sustained attention	57(2)	D	+2	No recommendation
	Memory	98(4)	D	+2	No recommendation
	Maths	32(1)	D	+2	No recommendation
	WM	133(5)	D	+2	No recommendation
	Logical reasoning	28(1)	D	+2	No recommendation
**Guarana**	**Mental Fatigue**				
	Info processing speed	130(1)	D	0	No recommendation
	WM	130(1)	D	0	No recommendation

	**Physical fatigue**				
	Info processing speed	40(1)	D	0	No recommendation
	Complex RT	40(1)	D	0	No recommendation
	Memory	40(1)	D	0	No recommendation
	WM	40(1)	D	0	No recommendation
	**None**				
	Memory	28(1)	C	0	No recommendation
	Logical reasoning	54(2)	D	0	No recommendation
**Prebiotics**	**None**				
	Info processing speed	29(1)	C	+1	No recommendation
	Attention	29(1)	C	+1	No recommendation
	Memory	29(1)	C	+1	No recommendation
	Inhibitory control	29(1)	C	+1	No recommendation
